# Topological Sholl descriptors for neuronal clustering and classification

**DOI:** 10.1371/journal.pcbi.1010229

**Published:** 2022-06-22

**Authors:** Reem Khalil, Sadok Kallel, Ahmad Farhat, Pawel Dlotko

**Affiliations:** 1 American University of Sharjah, Department of Biology Chemistry and Environmental Sciences, Sharjah, United Arab Emirates; 2 American University of Sharjah, Department of Mathematics, Sharjah, United Arab Emirates; 3 Dioscuri Centre in Topological Data Analysis, Mathematical Institute, Polish Academy of Sciences, Warsaw, Poland; Ernst-Strungmann-Institut, GERMANY

## Abstract

Neuronal morphology is a fundamental factor influencing information processing within neurons and networks. Dendritic morphology in particular can widely vary among cell classes, brain regions, and animal species. Thus, accurate quantitative descriptions allowing classification of large sets of neurons is essential for their structural and functional characterization. Current robust and unbiased computational methods that characterize groups of neurons are scarce. In this work, we introduce a novel technique to study dendritic morphology, complementing and advancing many of the existing techniques. Our approach is to conceptualize the notion of a Sholl descriptor and associate, for each morphological feature, and to each neuron, a function of the radial distance from the soma, taking values in a metric space. Functional distances give rise to pseudo-metrics on sets of neurons which are then used to perform the two distinct tasks of clustering and classification. To illustrate the use of Sholl descriptors, four datasets were retrieved from the large public repository https://neuromorpho.org/ comprising neuronal reconstructions from different species and brain regions. Sholl descriptors were subsequently computed, and standard clustering methods enhanced with detection and metric learning algorithms were then used to objectively cluster and classify each dataset. Importantly, our descriptors outperformed conventional morphometric techniques (L-Measure metrics) in several of the tested datasets. Therefore, we offer a novel and effective approach to the analysis of diverse neuronal cell types, and provide a toolkit for researchers to cluster and classify neurons.

## Author summary

Variations in the three-dimensional shape (i.e morphology) of neuronal populations reflect underlying functional differences. Thus, accurate description and classification of neuron morphology are essential for functional characterization. We introduce a new method for clustering and classification of neurons based on dendritic morphology, leveraging concepts from topological data analysis. In doing so, we develop mathematical descriptors that quantitatively and objectively assess structural differences among neuronal cell types and thus classify them. Each Sholl descriptor that is assigned to a neuron is a function of a distance from the soma with values in real numbers or more general metric spaces. We retrieved neuronal reconstructions from different animal species and brain regions using the large public repository https://neuromorpho.org/ for clustering and classification tasks. The results reveal that certain descriptors, thus key morphological features, can reliably differentiate particular cell classes in agreement with expert assignment. Given that our method discriminates among different cell types, as well as reveal subclasses of cells, its utility may be extended to other species and brain regions.

## 1 Introduction

Neuronal morphology dictates how information is processed within neurons [1], as well as how neurons communicate within networks [2]. Thus, given the large diversity in dendritic morphology within and across cell classes, quantifying variations in morphology becomes fundamental to elucidate neuronal function. Neurons in the mammalian cerebral cortex have traditionally been divided into two major groups: spiny and smooth neurons [3]. In the adult, spiny neurons are excitatory neurons, whereas smooth neurons are inhibitory neurons [4]. Furthermore, spiny neurons are typically classified according to the lamina where their soma is located and by dendritic morphologies. Consequently, this classification scheme allows for further subdivision and identification of pyramidal neurons and spiny stellate neurons. Pyramidal cells play a critical role in circuit structure and function, and are the most abundant type in the cerebral cortex (70–80% of the total neuronal population) [5]. The dendritic morphology of pyramidal cells can vary substantially among cortical areas within a species [6–9], across species [10, 11], and even across development [12–14]. Developmental changes in the dendritic morphology of pyramidal cells is mirrored by structural changes in connectivity [15–18].

Similarly, neocortical GABAergic interneurons are important in shaping cortical circuits, accounting for 10–30% of all cortical neurons [19, 20]. Classification of GABAergic interneurons has proved to be especially challenging due to their diverse morphological, electrophysiological, and molecular properties [4, 21]. Importantly, morphological differences among classes and subclasses of pyramidal cells and interneurons are presumed to be functionally relevant. Moreover, changes in dendritic morphology is thought to underlie various neurodevelopmental [22], and acquired [23–27] disorders. Thus, given the key role of pyramidal cells and interneurons in cortical function in health and disease, it is important to differentiate among their subclasses through objective descriptors and classification tools.

Neuronal morphological descriptors take as an input a digital reconstruction of a neuron. Standard descriptors, including L-measure metrics, return a real-valued measurement describing chosen spatial feature of such a reconstruction. Conventional Sholl analysis [28] associates a function which counts the number of dendritic crossings of a given sphere of radius *r* centered at the soma. In general, the obtained neuronal features are then used to quantitatively assess and cluster cell classes using standard supervised [21, 29, 30], and unsupervised [31–33] clustering algorithms.

In recent years, the field of computational topology has become increasingly more popular in the characterization of tree structures, including neurons. The value of a topological descriptor is a persistence diagram; a structure that potentially carries more information than conventional numerical invariants. For example, [34] developed a new algorithm called “Topological morphological descriptor”, or TMD, which is based on topological data analysis methods (i.e. persistence diagrams) to classify families of neurons. In a more recent study [35], the authors use TMD to classify cortical pyramidal cells in rat somatosensory cortex. The topological classification was largely in agreement with previously published expert assigned cell types. Furthermore, [36] present a framework based on persistence homology to compare and classify groups of neurons. An alternate technique to study neuronal morphology was presented in [37] which assesses neuronal arbor morphology through sequence representation.

The relative success of topological methods has encouraged us to develop new, more elaborate descriptors that can advance the current methods. The approach we suggest, and then pursue, is to view a morphological feature describing a neuron as a function of distance from the soma which takes values in some relevant metric space. This is effectively an extension of the original approach of Sholl [28] which is based on tracking changes in branching information as one moves away from the soma, and which proved quite successful in the field (see [38, 39]).

By choosing neurobiologically meaningful features such as wiring (total dendritic length), branching pattern, tortuosity, taper rate, root angle, and others, we can monitor the trajectory of dendrites as they radiate from the soma. Consequently, this enables us to extract more detailed information available in the neuronal reconstruction. A “Sholl Descriptor” is then a rule that associates to each neuron, and for a given feature, a function with values in the real numbers, or in the metric space of persistence diagrams or in even more general metric spaces. This association must be both isometry invariant and *stable* (details in the text). The advantage is that Sholl descriptors endow the set of isometric neurons with *pseudo-metrics*, and this is a very convenient way to tackle the problem since the closer the neurons are in the underlying descriptor metric, the more of this feature they share. This method provides an objective and interpretable tool to compare and analyze neuronal morphologies.

In this paper, we develop eight Sholl descriptors, representing eight morphological features. Our clustering results were consistently better in separating different neuronal cell types than clustering methods based on raw morphometric L-measure data. Certain descriptors result in complete separation of selected groups of neurons. Importantly, specific features that play a major role in differentiating a group of neurons can be identified.

## 2 Methods

In this study, we develop a toolkit of eight descriptors, labeled as *tortuosity, leaf index, flux, branching pattern, energy, wiring, Sholl-TMD and taper rate*, and then propose their implementation. We did not run the taper rate descriptor on the tested datasets since information about diameter of dendrites was not available. Therefore, only seven descriptors were used in this study.

**Fig 1:**
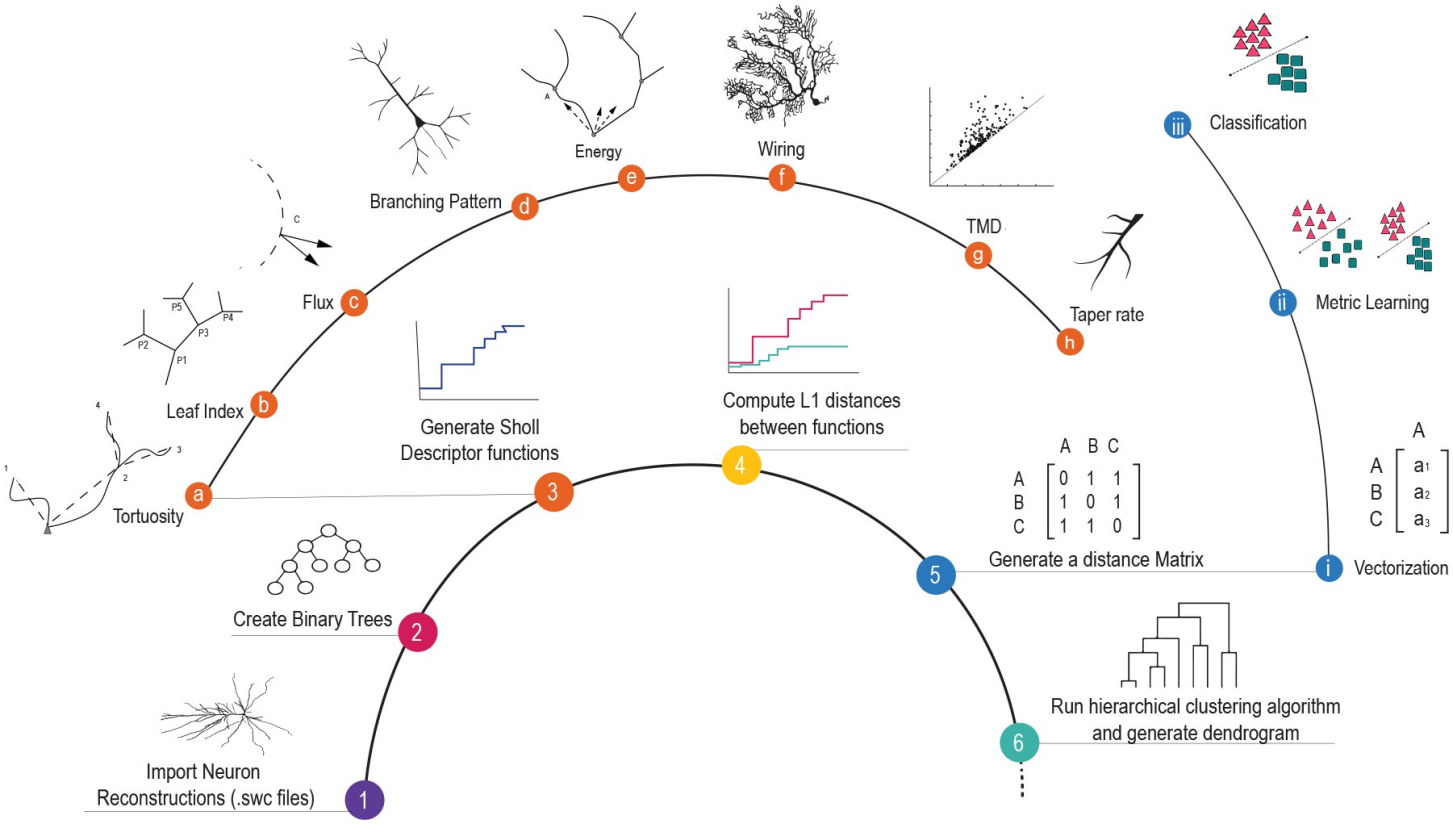
A process diagram to generate and cluster morphological data. Reconstructions of neuronal cell types are encoded in Sholl descriptors which are then used for clustering and classification. A representation of each Sholl descriptor is depicted in steps **a-h**. The process of clustering different neuronal cell types is shown in steps 1–6. Once neurons are vectorized based on descriptor metrics, we apply metric learning techniques to obtain classification (steps **i-iii)**.

The descriptors are used to differentiate among and classify different neuronal cell types using a wide range of methods; starting from dendrogram analysis to more advanced machine learning techniques (Fig 1). Given one of our eight morphological features, denoted by the Greek letter *ϕ*, we associate to such a feature a “Sholl descriptor” Φ which is a map from the set of neurons to a metric space of functions that is invariant under isometries and has stability properties (Definition 2.2). Each such Sholl descriptor Φ gives rise to a pseudo-metric *d*_*ϕ*_ on the set of all neurons §2. This (Sholl) *d*_*ϕ*_ measures proximity of neurons with respect to the particular feature *ϕ*. Specifically, the closer the neurons are under that metric, the more of the features captured by *ϕ* they share.

Sholl descriptors have to be combined in order to optimize both clustering and classification. We present three different methods of combining the descriptors, each with its own merits. The first combination method is unsupervised in that it does not depend on the way we subdivide our dataset into classes (C.2 in S1 Text). It is based on combining the different representations of each neuron by means of vectorization of its Sholl descriptor function. The second method combines distances between multiple features into a new metric. The combination is obtained by optimizing coefficients of a linear combination of distances by taking the coefficients of such a combination from an equi-spaced grid with the aim to maximize separation of classes (C.1 in S1 Text). If a reasonable combination cannot be produced, the classes are indistinguishable under all used morphological descriptors. The third combination method is a *supervised classification* technique which is achieved by means of metric learning.

In this criterion, a *detection algorithm* is introduced to measure the effectiveness of the considered features in differentiating groups of neurons (see §2.3 and D in S1 Text), and used to pre-select Sholl descriptors that are most effective in differentiating a given set of neurons (i.e. ones with highest detection rates). Those descriptors are then used to vectorize neurons. Once vectorized, a metric learning algorithm is applied to obtain an optimal Euclidean metric that can differentiate between the representation of the considered neurons. The second and third methods are checked for ‘overfitting’ (E.1 in S1 Text). The new metric obtained this way effectively reveals how close in features a random neuron is to the given classes.

### 2.1 Representation of neurons and terminology

We model a neuron *N* as a tree embedded in 3-dimensional space $R^{3}$. The tree is composed of a collection of rooted binary trees, all having a common root (the soma). Consequently, all branchpoints in a neuronal tree, with exception of the soma, have degree two. We assume that the root is located at the origin in the considered coordinate system. These rooted treed are also called the “primary” trees.

All 3D neuronal reconstructions used in this paper were acquired from the public repository NeuroMorpho.org [40]. The morphological structure of individual neurons is retrieved from an SWC file which contains a digital representation of the neuron as a tree structure that consists of points in $R^{3}$ joined by edges. Each marker has associated properties such as 1) its 3D spatial coordinates, 2) its radius denoting the thickness of the branch segment at a specific 3D location 3) a node type indicating whether it is soma, axon or dendrite, and 4) one parent marker to which it directly connects through neuronal arbors. The soma is always located at the origin of a reference frame.

**Fig 2:**
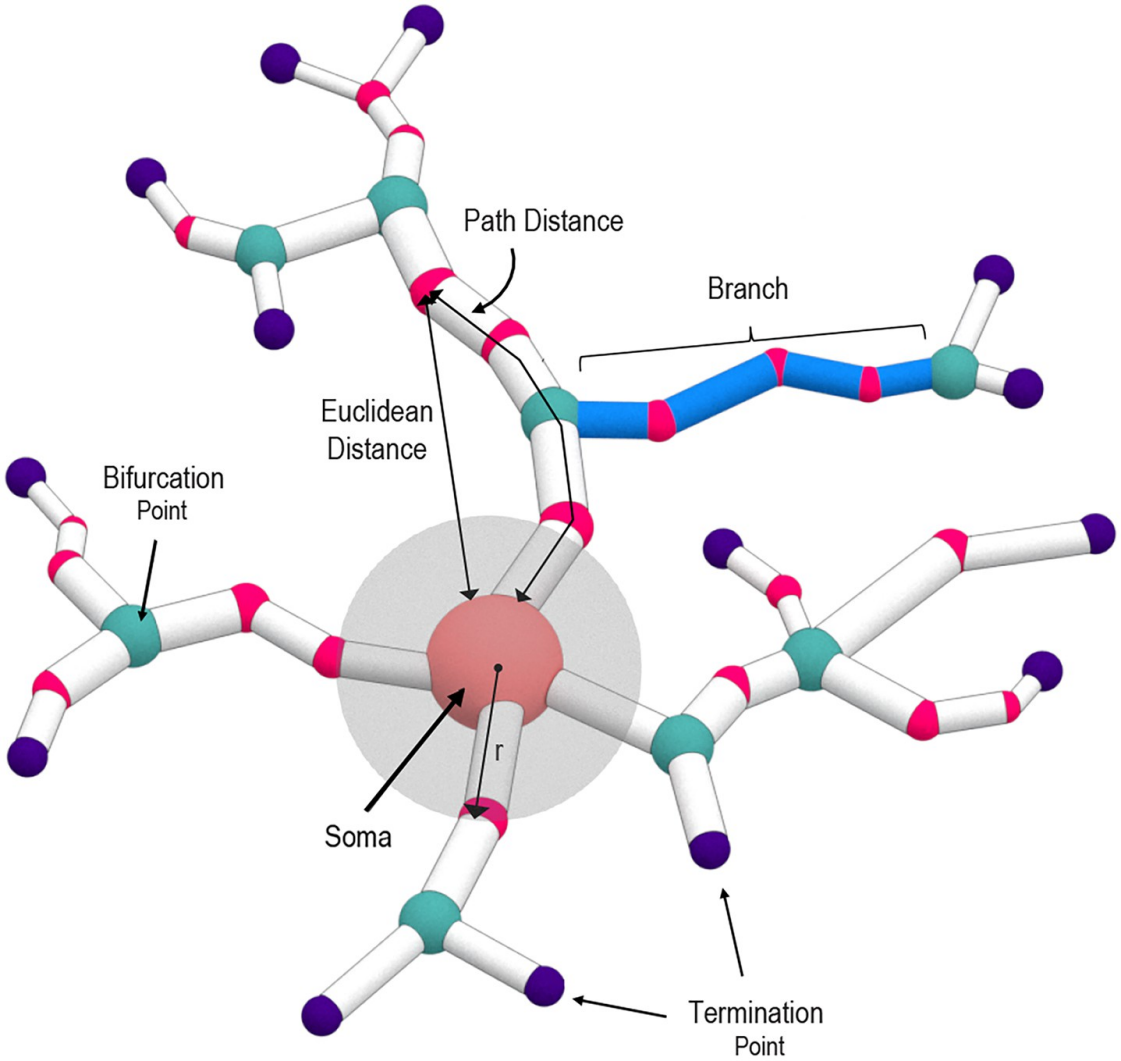
Representation of a neuronal tree structure. Schematic of a neuron illustrating key definitions used in the present work.

We will use the following notation and terminology, some of which are illustrated in (Fig 2):

- Capital letter *N* represents a neuron seen as a tree in 3-space.
- A class *C* of neurons is a set containing a selection of neurons of a particular type.
- A node in a neuron *N* is either the soma, bifurcation point, or a termination point.
  $$\begin{array}{c} \left\{\text{Nodes}\}=\{\text{Bifurcations}\}\cup \{\text{Soma}\}\cup \{\text{Terminations}\right\} \end{array}$$
  A branchpoint can be used interchangeably with bifurcation point. A leaf can be used interchangeably with a termination point.
- The number of terminal nodes in a tree is denoted as degree which is a proxy for tree complexity. The number of branches of a tree is twice the degree of that tree minus one, while the number of bifurcations is degree minus 1.
- Radial distance means Euclidean distance as measured from a point to the soma.
- Path distance is the distance from a point to the soma along a dendrite.
- A branch is a part of the dendrite that lies between two branchpoints, between one branchpoint and a termination point, or exiting the soma.
- Two nodes are parent-child related if they are adjacent on a branch. The node closer to the soma in path distance is called the parent, and the node farther away from the soma is called the child.
- A neuron has span *R*(*N*) if it can fit in a ball centered at the soma of radius *R*(*N*), and in no smaller ball. In practice and almost always, *R*(*N*) is the largest radial distance from soma to any of the nodes.
- *L*(*N*) is the length of the longest dendrite stemming from soma and ending at a termination point.
- A neuronal feature is denoted by the Greek letter *ϕ*. It is always topological or morphological in nature, and its associated (Sholl) descriptor will also be denoted by *ϕ*.
- A feature *ϕ* gives rise to a metric on the set of neurons which is denoted by *d*_*ϕ*_.

### 2.2 Sholl descriptors

Below we introduce Sholl descriptors and their basic constructions. All technical details appear in A and B in S1 Text.

**Definition 2.1** *A “Sholl descriptor” is any rule* Φ *that associates to a given neuron N (represented as a tree embedded in* $R^{3}$*) a compactly supported function* ${\tilde{\phi }}_{N}$ *whose independent variable is either path or radial distance from the soma, and whose values are in a metric space X. We further require this function to be both isometry invariant and stable with respect to reconstruction errors*.

All our Sholl functions are maps ${\tilde{\phi }}_{N}:I\to X$, for each given neuron $N\subset R^{3}$, where *I* is either the interval [0, *R*(*N*)] or [0, *L*(*N*)] (see §2.1 for definitions of *R*(*N*) and *L*(*N*)) and *X* is a metric space. To ensure that our constructions are independent of scale, the domains of all functions are normalized to an interval [0, 1], and the normalized Sholl function is written *ϕ*. The *ϕ* is stable as described in F in S1 Text. Isometry invariance means that if *N*′ is obtained from *N* by a linear isometry (i.e. a rotation or reflection by a plane passing through the soma), then ${\tilde{\phi }}_{N}$ and ${\tilde{\phi }}_{N^{\prime }}$ are identical functions.

The following are Sholl descriptors that we discuss in this paper.

**Fig 3:**
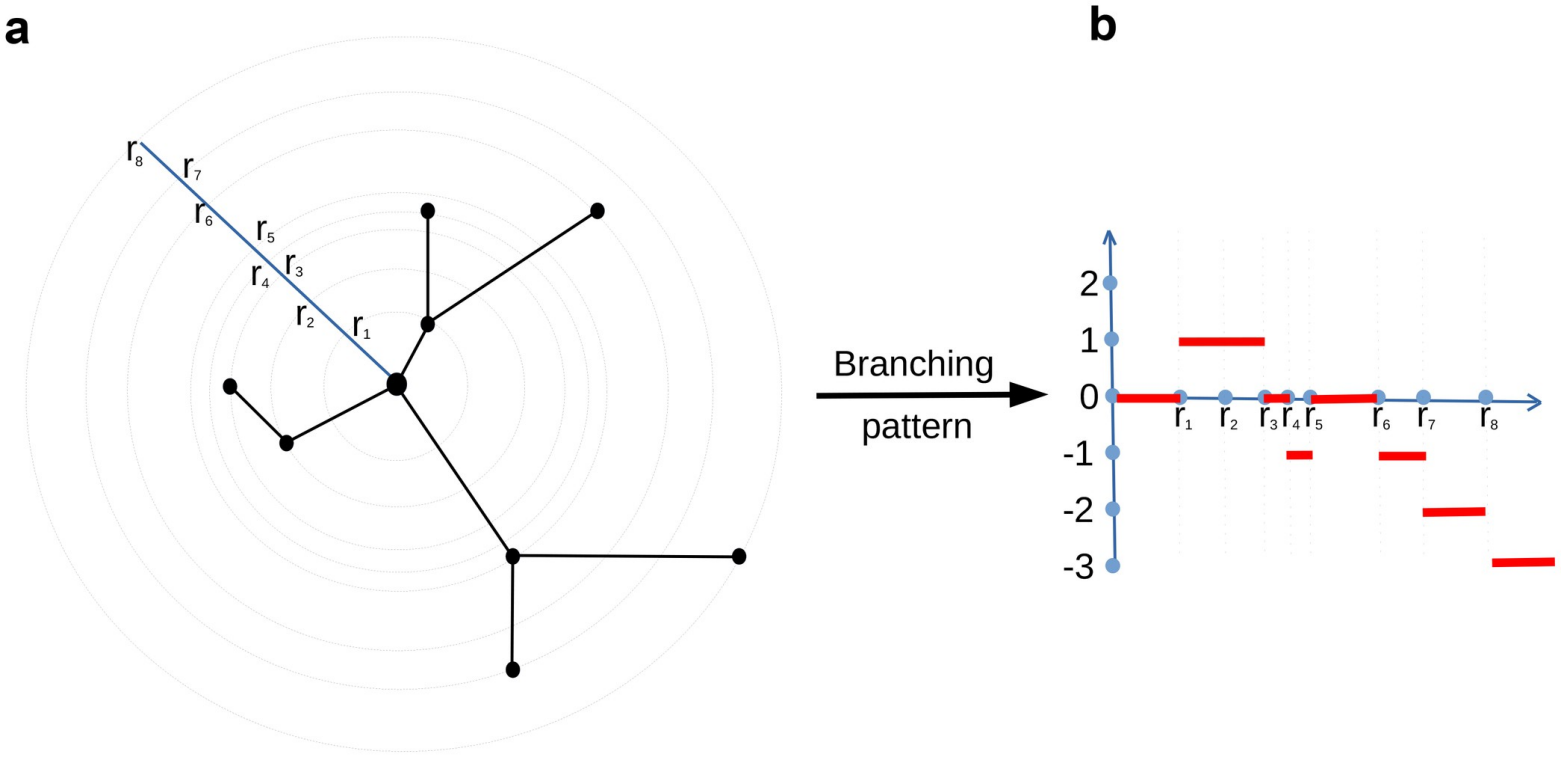
Sholl descriptor using branching pattern. (**a**) A neuron *T* embedded in $R^{2}$. The root of *T*, representing the soma, is located at the center of the disk. The concentric circles, centered at the root, are determined by the branch points they go through at distances *r*_1_, …, *r*_8_ from the soma. At radius *r*, the Sholl function *ϕ*_*T*_ (**b**) counts the number of nodes in a ball or a radius *r* centered at the root.

1. **Branching pattern**: This is an integer–valued descriptor equals the number of bifurcations minus the number of leaves in a distance *r* from the soma. As the radius *r* changes, this number evolves. This fundamental construction is presented in Fig 3, and the branching pattern function is depicted for a simple tree. The value of the descriptor for that neuron is the step function on the right. Note that a different (branching) Sholl descriptor is obtained when a radial distance is replaced with a path distance from the soma. The branching structure of a neuron is important for determining function. For example, in primary visual cortex, branching structure is related to orientation and direction selectivity of neurons.
2. **Tortuosity**: Tortuosity of a given path is defined as the ratio of the path length by the Euclidean distance of its endpoints. The tortuosity descriptor at a distance *r* from the soma measures the mean tortuosity of all branches connected to the soma within the sphere of a radius *r* centered at the soma. This descriptor informs about the dendrite’s growth mechanisms and its method of reaching synaptic targets.
3. **Flux**: This descriptor starts with a sphere of a radius *r* centered at the soma. For all the dendrites intersecting that sphere it associates to *r* the sum of the angles between dendrites and normal directions to the sphere. The flux construction can be viewed as a parameterized version of the root-angle construction in [41]. This construction associates to a leaf the angle between the radial normal and the main branch ending at that leaf. This measure quantifies centripetal bias which is based on the branching characteristics of dendrites.
4. **Taper Rate**: This construction is based on the width of dendritic segments at bifurcations, taken as a function of path distance to the soma. To be precise, the branching nodes are ordered according to their path distance to the soma. The value of the Taper Rate, for a radius *r*, is equal to the dendritic diameter at the closest node in a distance at most *r* from the soma (see B.2 in S1 Text). Dendritic tapering is a measure of the change in width along a dendritic segment from node to node. Dendritic tapering towards the terminal tips maximizes current transfer and thus leads to enhanced integration of synaptic inputs at different locations.
5. **Leaf Index**: This construction counts the number of leafs that can be reached from a given node using paths that goes away from the soma. If the node is taken to be the soma, this number is the total number of leaves in the tree. If the node is taken to be a leaf, the value is one (that leaf). The Leaf Index considers branching nodes as a function of radial distance. For a given value *r*, consider the last branchpoint *b* that is not farther away from soma than *r*. The value of Leaf Index at the radius *r* is equal to the number of leafs reachable from *b*. This measure quantifies centripetal bias which is based on the branching characteristics of dendrites
6. **Energy**. This descriptor is based on the idea of viewing the nodes as charged electrons that generate electric fields. Using the superposition principle, the resulting vector field at the soma is recorded. Its length is an isometry invariant quantity. For any given radius *r*, we can carry out the same construction using only those nodes not further away from soma than *r*, and take the sum of their contributions at the soma. The energy vector and strength give a measure of how the nodes are distributed relative to the soma. If we divide space in octants, with the soma at the origin, then the more nodes present in the same octant, the greater the energy. This is similar to caulescence which refers to a prominence of a main path in a tree structure to reveal tree asymmetry [42]. We can use the Energy vector very conveniently to identify polarized neurons. This is explained and illustrated in C.3 in S1 Text.
7. **Total Wiring**. Take a tree being a connected component of a neuron that contain soma, restricted to a ball of a radius *r*. This construction measures the total path length of this tree. This is the sum of the path distances of all dendritic segments and it is related to the notion of Arbor Density or Arbor Territory [43]. Greater total wiring dendritic length means greater invaded space which render the dendrites available to more incoming connections and greater connectivity.
8. **TMD**. This is the Topological Morphological Descriptor of [34] (see B.7 in S1 Text for details) redesigned to be a Sholl function. It assigns to a given value *r*, the TMD of the connected component of the tree, that contains the soma, intersected with a ball of a radius *r* centered in the root. Hence, the Sholl-TMD can be seen as a *r*-varying persistence diagram where the changes happens on a discrete set of *r*–values. The metric used in this case is the integral of Wasserstein metric *W*^*p*^. More specifically, suppose we want to compute the difference between two Sholl-TMD functions *f*_1_ and *f*_2_ where *f*_1_ is changing on points $r_{1}^{1},\ldots ,r_{n}^{1}$ and *f*_2_ is changing on $r_{1}^{2},\ldots ,r_{k}^{2}$. To compute the distance, we take *r*_1_, …, *r*_*n*+*k*_ being the union of $r_{1}^{1},\ldots ,r_{n}^{1}$ and $r_{1}^{2},\ldots ,r_{l}^{2}$. Then the distance between *f*_1_ and *f*_2_ is then given by
  $$\sum_{i=1}^{n+k}W^{p}\left(f_{1}\left(r_{i}\right),f_{2}\left(r_{i}\right)\right)$$

**Fig 4:**
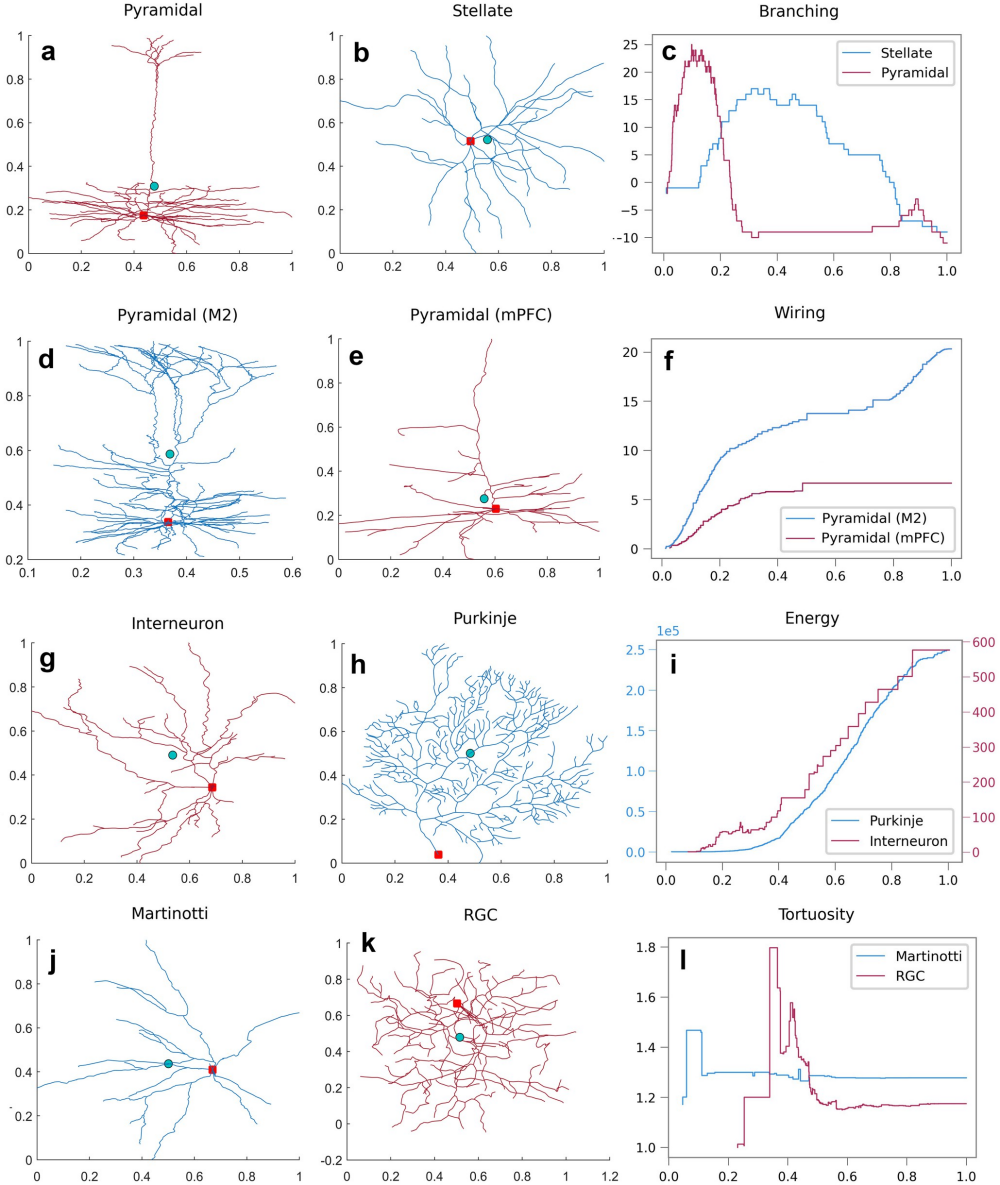
Representative neurons and their corresponding step functions. (**a**) Pyramidal and (**b**) stellate cell. (**c**) The step functions for each neuron generated from the branching pattern Sholl descriptor. The branching Sholl functions show that neuron (**a**) is branching quickly near the soma, and leaves appear much closer to the soma than for neuron (**b**). The number of primary branches for each neuron is the value of the corresponding function at 1. (**d**) Pyramidal cell in secondary motor cortex (M2) and (**e**) in medial prefrontal cortex (mPFC). (**f**) The step functions for neurons (**d**) and (**e**) generated from the wiring Sholl descriptor. The sharp increase towards the end for the wiring Sholl function for cell **d**. reveals the existence of an apical tuft. (**g**) Interneuron and (**h**) Purkinje cell. (**i**) The step functions for each neuron generated from the energy Sholl descriptor. (**j**) Martinotti and (**k**) Retinal ganglion cell (RGC). (**l**) The step functions for each neuron generated from the tortuosity Sholl descriptor. The red dot represents the soma and the turquoise dot is the barycenter of all nodes.

**Example 2.2** In Fig 4, Tree structures of two different neurons are chosen (A) pyramidal and (B) stellate. The corresponding Sholl descriptor functions reveal obvious difference (C). The red curve which depicts the branching pattern of the pyramidal cell reveals that branching occurs rapidly close to the soma, but much slower as you move further away from the soma. Conversely, branching for the stellate cell is changing uniformly and steadily as you move away from the soma.

### 2.3 Sholl analysis

Sholl Descriptors can be used to cluster and classify datasets of neurons. In this paper, we use them combined with standard hierarchical clustering methods, a detection algorithm (introduced in this paper, used for feature selection), grid search, and metric learning functions. Note that this is just an example of the used-cases for Sholl descriptors. All technical details associated to this section are deferred to S1 Text. In this paper, we present the following analysis:

1. Analysis based on a single descriptor:
  - (Clustering) Given a dataset of unlabeled neurons we can use a particular descriptor in order to cluster them according to that descriptor. For example, if *ϕ* = *T* is tortuosity, we can set the distance matrix for the associated Sholl pseudo-metric *d*_*T*_ and then run standard hierarchical clustering to obtain dendrograms. The obtained dendrogram reveals whether neuronal cell types differ according to their tortuosity (i.e. cluster together), or if their tortuosity is comparable (cells from different neuron types will be intermingled).
  - (Detection) It is a simple distance-based measure designed for the purpose of this paper. It assesses the performance of a given descriptor in identifying neurons with a given label within a dataset. Its idea is to find a ball *B* that contains at least *p*% of all neurons with the label *l* and, in addition, the neurons of the label *l* compose at least *p*% of all neurons in *B*. Detection rates can be used as feature selection when running classification schemes (see Table 1).
2. Analysis based on a combination of descriptors:
  - (Vectorization in unsupervised setting C.2 in S1 Text). A given neuron can be converted into a vector using our descriptor functions. Once neurons are converted into vectors, standard clustering and regression algorithms can be applied to them.
  - (Combination with weights C.1 in S1 Text). By linearly combining metrics originating from various descriptors, new clustering metric that separates neurons of different types can be obtained. More precisely, given a collection of neurons *N*_1_, …, *N*_*k*_ with labels *l*_1_, …, *l*_*k*_ we compute a number of distance matrices *M*_1_, …, *M*_*n*_. The matrix *M*_*i*_ is a distance of *ϕ*_*i*_ descriptors of the considered neurons. In this method a joint distance matrix *M* = *α*_1_ *M*_1_ + *α*_2_ *M*_2_ + … + *α*_*n*_ *M*_*n*_ is constructed. The coefficients *α*_1_.…, *α*_*n*_ are chosen to maximize the combined distance between neurons of different labels, and minimize distance of those with the same label.
  - (Classification) Given a dataset of neurons distributed among a number of classes, and given a number of morphological features, we can determine with which class a newly introduced neuron is associated; that is, with which class it shares the most morphological features. More precisely, suppose we are given classes of neurons *C*_*i*_, 1 ≤ *i* ≤ *n*, and morphological descriptors *ϕ*_*j*_, 1 ≤ *j* ≤ *k*, which can be measured for all neurons. Given a newly introduced neuron *N*, we can determine with measurable likelihood the class *C*_*i*_ closer to *N* in the pseudo-metrics $d_{\phi_{1}},\ldots ,d_{\phi_{k}}$. To that end, we turn neurons into vectors, as described in C.2 in S1 Text and then devise an optimal Euclidean metric *D*_*ML*_, out of the $d_{\phi_{j}}$’s using a metric learning algorithm [44]. The optimality here means that *D*_*ML*_ maximizes distance between neurons in different classes, and minimizes it between neurons of the same class. The smallest *d*_*N*_ distance between *N* and the given classes indicates which class the neuron should belong to.

**Table 1:** Detection rates applied to all datasets based on all descriptors. Numbers represent percentages. Green represents complete detection of a class (90%-100%), while pink represents detection rates between 80%-89%.

Set	Description	Energy	Flux	Leaf index	Branching	Wiring	Tortuosity	TMD Sholl	TMD classical
1	retinal ganglion (48)	75	85	100	88	94	96	71	77
	hippocampal pyramidal (45)	64	69	43	71	71	93	82	84
	somatosensory spiny stellate (43)	76	67	98	71	72	91	63	61
	primary visual Martinotti (20)	40	48	95	45	30	100	60	55
2	cerebellar purkinje (60)	93	83	63	92	69	92	77	77
	primary visual interneuron (66)	44	99	79	96	78	77	94	94
	hippocampal granule (77)	60	84	84	96	84	87	62	62
	spinal cord interneuron (77)	40	74	73	84	51	68	76	78
3	somatosensory pyramidal (20)	40	100	60	95	75	70	90	90
	medial prefrontal pyramidal (46)	80	98	100	96	77	93	85	89
	secondary motor pyramidal (30)	65	90	74	83	94	82	63	73
4	human hippocampal pyramidal (54)	57	85	87	78	89	83	81	75
	mouse hippocampal pyramidal (50)	63	96	53	90	96	88	96	94

## 3 Results

We propose seven descriptors based on the morphological features listed above. A schematic of each descriptor is illustrated in steps a-h in Fig 1, and all terms are defined in §2.2. We illustrated the discriminative accuracy of our Sholl descriptors on four datasets, providing evidence that relevant Sholl descriptors can reliably discriminate among different classes of neurons in agreement with previously published assignment. The tested datasets were chosen to cover diverse types and subtypes of neurons across different regions and animal species.

### 3.1 Distinguishing among classes based on a single feature

**Fig 5:**
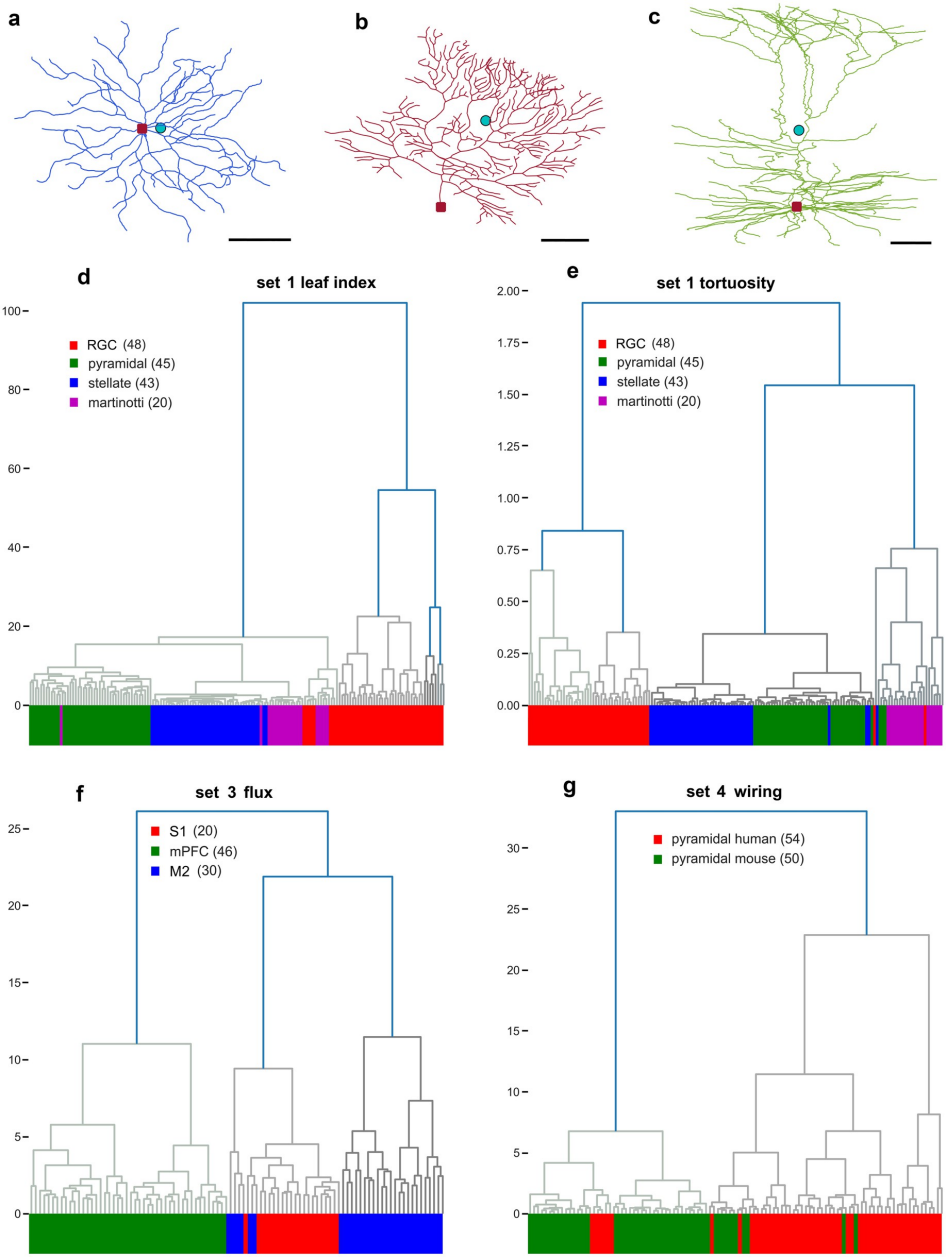
Hierarchical clustering trees for selected descriptors. Representative neuronal reconstructions of (**a**) interneuron (**b**) purkinje, and (**c**) cortical pyramidal cell, in blue, magenta, and green colors respectively. Representative dendrograms based on descriptors for (**d**) Leaf index and (**e**) tortuosity for Dataset 1, (**f**) Flux for Dataset 3, and (**g**) wiring for Dataset 4. For the reconstructions (**a,b,c**), the red dot represents the soma and the turquoise dot is the barycenter of all nodes. Scale bar in a = 10 *μ* M, b = 25 *μ* M, and c = 50 *μ* M.

The first two datasets that we analyzed consisted of neuronal classes that appear visually different to the naked eye. The aim was to test the discriminative power of individual Sholl descriptors in differentiating easily discernible cell types, and thus cluster them. A representative neuron type from each dataset is shown in Fig 5a–5c. We applied our Sholl descriptors on **Dataset 1**, comprising four neuronal cell types in the mouse brain: retinal ganglion cells (n = 48) [45], pyramidal cells (n = 45) [46], spiny stellate cells (n = 43) [47], and Martinotti cells (n = 20) [48]. Seven different Sholl functions were computed on this set (all but taper-rate since the information about diameter of dendrites was not available) and distance matrices between neurons were computed for each Sholl function.

Table 1 reports the detection rates (see §2.3 and D in S1 Text) for all descriptor functions for all datasets. For convenience, we briefly explain how to read the table: detection rates are correlated to standard clustering dendrograms. If a rate of 90% appears as an entry corresponding to a class of neurons and a particular feature *ϕ*, this means that 90% of all neurons in that class are within a ball in the *d*_*ϕ*_ metric, and within that ball, less than 10% of neurons are outside that class. Detection rates highlighted in pink are above 80% while rates of 90% and higher are highlighted in green. Therefore, we say a class has been *detected* by a descriptor *ϕ* if the rate of detection for that class is at least 90%.

As shown in this table, the performance of all descriptors was high in that each class was completely detected by at least two descriptors. Remarkably, the leaf index and tortuosity descriptors detected three and four classes respectively, suggesting that these descriptors alone are sufficient in differentiating among neuronal types in this particular dataset. Retinal ganglion cells was detected by five out of seven Sholl descriptors. The dendrogram based on the leaf index and tortuosity descriptors are shown in Fig 5d and 5e respectively. The horizontal axis of the dendrogram represents all the neurons in this dataset while the vertical axis represents the distance between clusters. Interestingly, pyramidal and stellate cells each formed a distinct cluster in the leaf index dendrogram (Fig 5d), while retinal ganglion cells and spiny stellate cells each formed their own cluster in the tortuosity dendrogram (Fig 5e). The fact that retinal ganglion cells were fully detected by five out of seven descriptors Table 1, suggests that this cell type is characterized by a unique set of morphological features.

To validate this finding and illustrate the consistency in performance of our Sholl descriptor, we included **Dataset 2**, again testing whether individual descriptors can differentiate among very different cell types. This dataset consisted of mouse cerebellar purkinji cells (n = 60) [49], primary visual cortical interneurons (n = 66) [50], hippocampal granule (n = 77) [51], and spinal cord interneurons (n = 77) [52]. Table 1 reveals that the branching and flux descriptors yielded high detection rates. Furthermore, the performance of the TMD Sholl descriptor was comparable to that of the classical version in detecting all cell classes, both of which returned a high detection rate for the cortical interneurons (detection = 94%). In contrast, the wiring and leaf index descriptors performed poorly in separating three of the four neuronal cell types, as evidenced by the low detection rates. This suggests that these particular morphological features are largely uniform across these cell types, while other descriptors allow for better separation of the considered classes of neurons.

### 3.2 Subclustering within a neuronal class

To ensure sufficient coverage of neuron types, we also tested our Sholl descriptors in clustering pyramidal cells from different cortical areas in the rat brain. Therefore, **Dataset 3** consisted of rat pyramidal cells in layer 5 of somatosensory (S1) (n = 20) [53], secondary motor cortex (M2) (n = 30) [54], and medial prefrontal cortex (mPFC) (n = 46) [55]. The discriminative accuracy in separating the three neuron types with many of the descriptor functions was very high. For example, the cluster analysis based on the flux descriptor shown in the dendrogram in Fig 5f resulted in nearly perfect clustering. The detection rate was highest in both medial prefrontal (98% detection) and somatosensory cortex (100% detection), followed by secondary motor cortex (90% detection). Likewise, the branching pattern, tortuosity, and TMD Sholl descriptors performed equally well as shown in (Table 1). Interestingly, the majority of pyramidal cells in secondary motor cortex formed their own distinct cluster, while several of these cells were clustered with other pyramidal cells in primary somatosensory cortex Fig 5f. This suggests the existence of two sub-populations of pyramidal cells in secondary motor cortex. Indeed, when we visually examined these neurons, we found striking similarities in morphology with pyramidal cells in primary somatosensory cortex. Similarity in morphology between cells in primary somatosensory cortex and secondary motor cortex could be due to proximity of these two cortical areas, as variation in neuronal morphology has been shown to be less pronounced between cortical areas that are close. For example, in the chimpanzee neocortex, total dendritic length, segment count, as well as spine number of pyramidal cells do not differ significantly between primary somatosensory cortex (area 3b) and primary motor cortex (area 4) [56]. Additionally, [57] showed that pyramidal cells in two proximal cortical areas in the mouse brain (secondary somatosensory cortex S2 and secondary motor cortex M2) are comparable in terms of total dendritic nodes (bifurcations and endings), and total dendritic endings. We note that there is also evidence to suggest major differences in pyramidal cell morphology even within a single cortical area [58]. Whether differences in pyramidal cell morphology in close cortical areas is more pronounced or subtle could have evolved to serve species specific adaptive behavior.

**Dataset 4** comprises reconstructions of hippocampal CA1 pyramidal cells taken from human (n = 54) and mouse (n = 50) [46]. In this study, the authors document important structural differences with respect to several morphometric features between the two species. For example, they show that human hippocampal CA1 pyramidal cells have double the number of stems (primary) basal dendrites and double the number of basal terminal endings. Additionally, human pyramidal cells have longer dendritic segments than in mouse. Therefore, as a proof of concept we sought to recapitulate these findings by running our descriptors on this dataset. We expected to at least cluster pyramidal neurons based on the branching pattern and wiring descriptors. Remarkably, all seven descriptors resulted in excellent detection of mouse pyramidal cells, with the exception of the leaf index descriptor. However, the leaf index descriptor returned very good detection of human pyramidal cells (87%). Furthermore, detection rates were generally higher for the mouse pyramidal cells versus the human pyramidal cells (Table 1). Indeed, the dendrogram in Fig 5g based on the wiring descriptor reveals two distinct clusters with excellent separation of mouse and human pyramidal cells. There was however some intermingling of neurons between the two groups. Similarly, as seen in (Table 1) the branching pattern descriptor resulted in excellent detection of mouse pyramidal cells (90%), while this descriptor returned reasonable detection of human pyramidal cells (89%). The TMD Sholl also proved to be effective in detecting mouse (96%) and (81%) human pyramidal cells, performing slightly better than the Classical TMD. Taken together, our findings are commensurate with the structural differences previously shown in [46]. Importantly, we illustrate that using a single descriptor can result in effective separation of pyramidal cells from different species.

### 3.3 L-measure metrics versus Sholl descriptors

**Fig 6:**
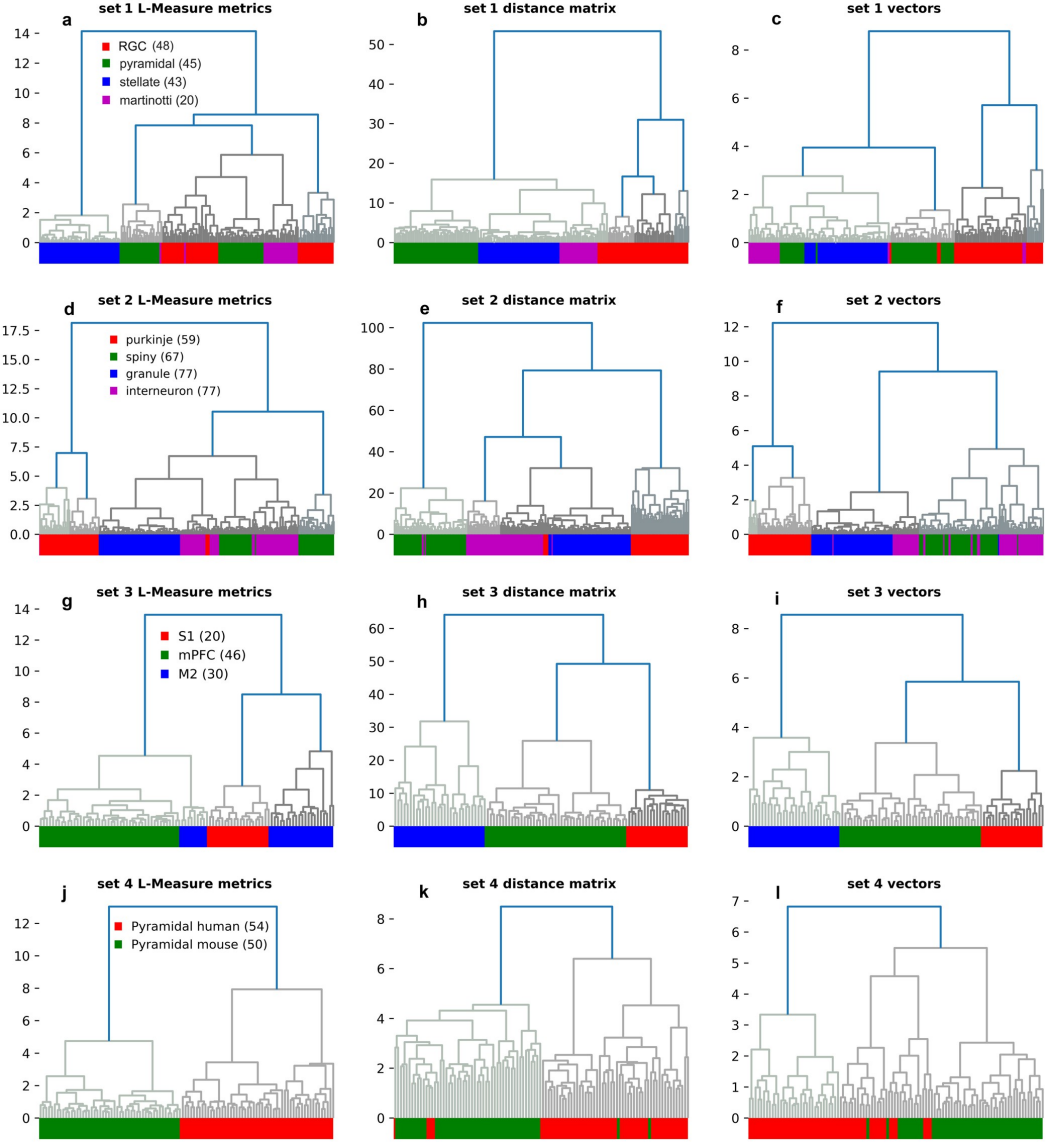
Hierarchical clustering trees comparing the L-Measure method, our combined Sholl descriptors, and our vector for all datasets. Representative dendrograms for morphological parameters extracted from (**a**) L-measure software, (**b**) Combined Sholl descriptors, and (**c**) our vector for dataset 1. Dendrograms for dataset 2 are presented in (**d,e,f**), dataset 3 (**g,h,i**), and dataset 4 (**j,k,l**). The “distance matrix” clustering is based on the distance matrix of the combined Sholl pseudo-metrics C.1 in S1 Text, while “vector clustering” is based on our unsupervized vectorization C.2 in S1 Text and Euclidean metric.

Given that individual Sholl descriptors performed well distinguishing certain neuronal classes, we then combined our descriptors in order to optimize clustering and to compare our methods to standard L-Measure metrics in separating neuronal classes. We ran two clustering algorithms. We first combined the metrics associated to our seven descriptors into an optimal one and setup its distance matrix. Then, for each given neuron, we extracted meaningful parameters from the Sholl functions and vectorized them as detailed in C.2 in S1 Text. A hierarchical cluster analysis algorithm was then applied using Ward’s linkage method and the Euclidean distance metric. Next, for each given neuron, we extracted all 43 morphological parameters available in the L-Measure software [59], combined them into a vector, and applied the same cluster analysis algorithm. Our clustering results for all datasets are presented in Fig 6. Neurons are color coded according to morphological type.

In **Dataset 1**, cluster analysis based on the L-Measure method returned six clusters with some intermingling of neurons from the four types Fig 6a, whereas analysis based on features captured by the Sholl descriptors resulted in clear separation of classes into four clusters Fig 6b. Additionally, pyramidal cells and retinal ganglion cells were found to populate two distant clusters with the L-measure method, while they constituted a single cluster in the dendrogram using our Sholl descriptors Fig 6b. Lastly, our vector method yielded reasonable separation of classes Fig 6c with slightly worse performance than our combined method, but remained somewhat better than the L-Measure method. Similarly, in **Dataset 2**, cluster analysis based on features captured by the Sholl descriptors resulted in excellent discrimination of classes comprising four clusters Fig 6e with some intermingling of neurons. However, clustering based on the L-Measure methods revealed two separate clusters for the spiny neurons and spinal cord interneurons Fig 6d. Moreover, some of the purkinji cells were clustered with the spinal cord interneurons. Clustering based on our vector returned reasonable separation of classes Fig 6f, with slightly poorer performance than our distance matrix.

For **Dataset 3**, the L-Measure method performed very well in separating the three classes albeit with two separate clusters of secondary motor cortex pyramidal cells Fig 6g. Cluster analysis based on our Sholl descriptors returned complete separation of classes Fig 6h, which was commensurate with results from our vector Fig 6i. Lastly, for dataset **Dataset 4**, the L-Measure method performed optimally in separating this dataset into two distinct clusters Fig 6j. Likewise, our Sholl descriptors discriminated pyramidal cells in humans from that in mouse with some intermingling from the two Fig 6k. Our clustering method based on our vector performed slightly better, but still clustered a few of the human pyramidal cells with the mouse ones Fig 6l. Taken together, the results demonstrate that our combined descriptor method and vectorization yield very good separation of classes, in some instances performing better than conventional methods in clustering different neuronal cell types. It is important to note that the L-measure vector is in $R^{43}$ while our vector is in $R^{24}$.

### 3.4 Classification and metric learning

**Fig 7:**
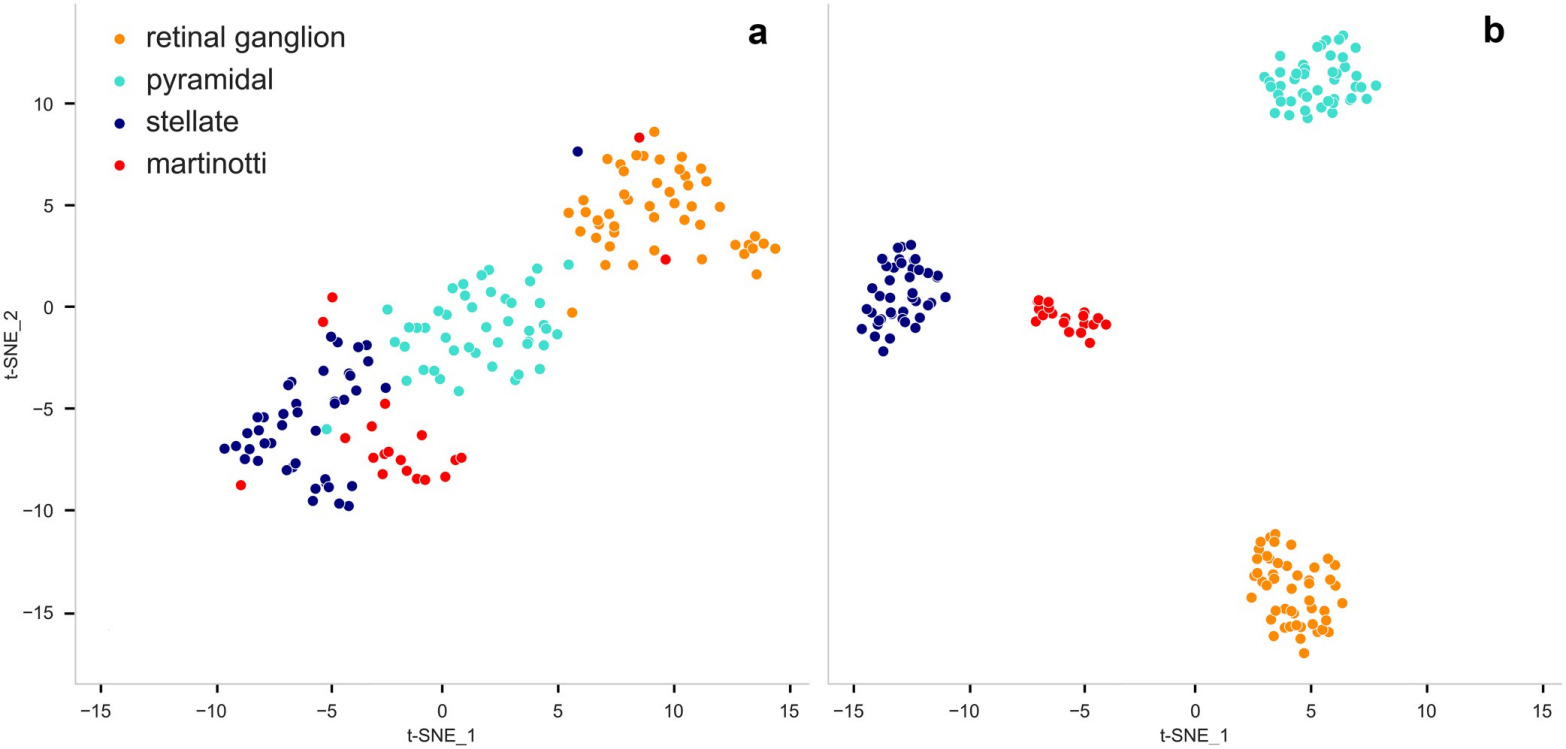
Classification and Metric learning for Dataset 1. Each dot represents a neuron which is color coded according to its class. tSNE was performed to reduce dimensions. (**a**) Vectorized neurons in Euclidean space. (**b**) Large Margin Nearest Neighbor (LMNN) classification for metric learning classification in the new metric space.

We applied our descriptors to **Dataset 1** comprising a total of 156 neurons from four neuronal cell types in the mouse brain in order to classify them (retinal ganglion cells (n = 48), pyramidal cells (n = 45), spiny stellate cells (n = 43), and Martinotti cells (n = 20)). We applied all our Sholl descriptors on this dataset (energy, flux, leaf index, branching, wiring, tortuosity, and Sholl TMD). Next, we vectorized the neurons (C.2 in S1 Text) based on these descriptors, resulting in classes of vectors in Euclidean space (in $R^{24}$). We then used a t-SNE plot to visualize our high dimensional descriptor vectors in 2D space (Fig 7a). Some neurons from different classes in this dataset are clearly overlapping and poorly separated. The data was then fitted and transformed into a new metric space which learns a Mahalanobis distance metric in the K-Nearest Neighbor (KNN) classification setting [44] (see E in S1 Text). Fig 7b displays the improved t-SNE plot which shows clear separation of the classes.

Classification models were built for all datasets using the following technique. In order to cross validate the model and to prevent overfitting 70% of data is randomly selected for the training phase and the rest is used for test. In order to test for overfitting we used repeated stratified k-fold cross validation with 10 folds technique where the training set is split into k equal sets or folds. The stratified k-fold ensures that we have the same number of samples for each class. Each k fold is used as a validation set and the k-1 remaining folds are used for training. A k-Nearest Neighbors (KNN) classifier is used in all models. Accuracy and Standard Deviations for the models in all datasets are: dataset1: 92.893 (6.596), dataset2: 94.022 (3.190), dataset3: 99.667 (2.333), dataset4: 98.294 (2.737).

## 4 Discussion

In this work we introduced novel descriptor functions of tree structures and applied them for classification and clustering of neuronal cell types. Notably, our methods complement and advance many of the existing techniques currently used to study neuronal morphology. Effectively, we construct a function with values in some metric space (Sholl function) for every neuron. This Sholl function captures the evolution of a particular morphological feature as distance from the soma increases. Sholl functions of different neurons can be compared and the distance between them informs of how similar given neurons are with respect to a particular morphological feature captured by the applied Sholl functions. We illustrate that certain descriptor functions can effectively cluster classes of neurons with subtle morphological variations, as well as discriminate among widely different classes of neurons in agreement with expert assignment. Additionally, we provide evidence that a single descriptor function can perform better in separating particular neuronal cell types than conventional L-Measure metrics, with enhanced performance using our combined descriptor metrics. Lastly, we leverage metric learning techniques to provide more robust classification. Our framework is powerful enough to separate diverse classes of neurons across different brain regions and species. Our results reveal several key findings regarding the considered datasets.

The four representative datasets used in this study, derived from different areas, layers, and species, were chosen to ensure morphological diversity and prove wide applicability of the presented techniques. In Dataset 1 (different types of neurons taken from different regions of the mouse brain), we show that the leaf index and tortuosity descriptors performed very well in recovering the clusters corresponding to neurons from different brain regions. Specifically, based on the tortuosity descriptor the Martinotti and pyramidal cells each formed their own cluster Fig 5e. Interestingly, dendritic tortuosity has been shown to vary among different non-pyramidal neuron classes in the rat brain, whereby Martinotti cells in layer II/II and V of the frontal cortex have higher tortuosity than other cell types [60]. In the mouse brain, dendritic tortuosity increases as a function of increasing branch order on apical dendrites of hippocampal CA1 pyramidal cells [61]. Additionally, dendritic tortuosity of layer II/III pyramidal cells appears to increase from caudal to rostral regions in mouse cortex [62]. Our tortuosity descriptor is therefore robust in detecting subtle differences in dendritic tortuosity among neuron groups. The upper limit for tortuosity values appears to be 2, which is consistent with prior reports [60]. Importantly, we improved discrimination accuracy by using a combination of descriptors which effectively assigns weights to the function with the best separation results. As shown in Fig 6b, the combination of descriptors resulted in complete separation of neuron types in this dataset.

Anatomical studies have shown that interneuron morphology is highly diverse in the cerebral cortex. For example, interneurons with similar somatodendritic morphology may differ in axonal arborization patterns [21]. Therefore, axonal morphometric features are typically required for accurate classification of interneurons as they have been shown to capture important differences among interneuron subtypes [63]. We did not analyze axonal features using our descriptors. However, based on dendritic features alone some of our descriptors (leaf index, tortuosity, and combined) were able to reliably distinguish interneuron subtype (Martinotti) from other neuronal cells types such as purkinje, retinal ganglion cells, and pyramidal cells (Dataset 1). Given the challenge associated with distinguishing among interneuron subtypes, we were intrigued as to whether our descriptors could separate a dataset comprised of only interneuron subtypes. Therefore, we performed additional analysis of a dataset comprising basket, aspiny, and bipolar cells. We found that the branching pattern descriptor worked well in distinguishing the cortical bipolar cells from the aspiny and basket cells. Likewise, the leaf index descriptor singled out the basket cells from the aspiny and the bipolar cells. The combined descriptor resulted in better separation of the three neuron types, albeit with some intermingling of neurons between the groups. In general, since the descriptors were designed for analysis of dendritic morphology, loss of axonal information in the analysis was an important constraint in the complete separation of the interneuron subtypes. Therefore, in a future study, we will focus our efforts on developing descriptors to incorporate important axonal features for a more robust classification scheme.

Collectively, the results highlight the robust nature of our framework in quantitatively characterizing and discriminating among different neuronal cell types. Certain morphological features and thus specific descriptors are better suited in separating distinct neuronal cell types. For instance, the branching pattern descriptor appears to perform very well in detecting most neuronal cell types. Conversely, the energy descriptor, which reveals the distribution of nodes around the soma, appears to reliably detect retinal ganglion cells, purkinje, and interneurons. Importantly, our use of metric learning techniques resulted in more optimal classification (Fig 7). Progress in the development of unbiased clustering methods to distinguish among groups of neurons will further our understanding of the relationship between brain structure and function. The toolkit of morphological descriptors introduced here, and the development of new methods will potentially lead to the discovery of novel sub-classes of neurons [64]. Additionally, our descriptors will aid efforts to uncover differences between normal and aberrant neuron morphology which is commonly associated with various disease states. For instance, changes in dendritic morphology have previously been described in a number of disease states, including Alzheimer’s disease [23], schizophrenia [65], and mental retardation [66]. Revealing morphological aberrations resulting from neurodevelopmental and acquired disorders is an important step in understanding the pathophysiology of these diseases. However, rigorous methods are needed to detect potential subtle differences between normal and aberrant neuron morphology. Therefore, given that our tool kit of descriptors discriminated among different types of cells as well as revealing subclasses of cells, its utility may be extended to the study of brain diseases potentially identifying which subtypes may be affected in various disease states.

In future work we will refine the functional metrics on Sholl descriptors, and further conceptualize and rigorously develop the notion of morphological versus morphometric descriptors (i.e. shape versus size). What is important in clustering and classification is not only to differentiate between groups of neurons, but also to identify which features are different. Furthermore, ongoing work will integrate several descriptors in a toolkit, making it easily retrievable and usable by researchers in the field. Since all the required neuron information is currently available from the SWC-files, our toolkit would be a useful companion for neuromorpho.org. Finally, and importantly, researchers looking to cluster and classify neurons can conveniently find our source codes and descriptors in a github repository, and can use them to analyse both the Sholl and the L-measure descriptors. We made all material accessible, and further tools will be added online as our work progresses.

## Supporting information

S1 Fig TMD as a Sholl-type descriptor. (**a**) Example of TMD-path decomposition on a simple planar tree. The soma marked with 1 is the root. Equicentered circles reveal the distances of nodes from the root. The furthest node is node 8. The paths from the TMD-path decomposition are: {[5, 4, 2], [3, 2, 1], [8, 6, 1], [7, 6]} (**b**), The tree *T* with a single path *x* starting at the root *R*. When using TMD as a Sholl-type descriptor by considering TMD of *T* ∩ *B*(*R*, *r*) we will only see the final barcode [0, *d*(*R*, 6)] for *r* ≥ *d*(*R*, 4). For the radii *r* between *d*(*R*, 6) and *d*(*R*, 4) the endpoint of the persistence interval will be equal *r*. When *r* reaches *d*(*R*, 4) the endpoint of the persistence interval it will then jump down to *d*(*R*, 6).
(TIF)

S2 Fig Dendrogram of a bipolar interneuron. A representative bipolar cell is shown in **(a)**. The “energy angle matrix” is used to separate the nodes into two clusters as shown in **(b)**. The dendrogram can be read as follows: the angles between pairwise energy vectors associated to red nodes are small as is for the blue nodes. However, the angles between energy vectors of blue and red nodes are much larger.
(TIF)

S3 Fig Detection rate method. Method used to determine detection rate. Each circle is the boundary of a disk in the Euclidean metric.
(TIF)

S4 Fig Representation of isomorphic trees. Representative isomorphic trees with entirely different (**a**) branching pattern and (**b**) tortuosity.
(TIF)

S5 Fig Representative trees in the Hausdorff metric. Representative tree (**a**) and similar trees (**b**) and (**c**) that are close to tree (**a**) in the Hausdorff metric.
(TIF)

S6 Fig Instability. Instability behavior for tortuosity descriptor.
(TIF)

S1 Text Supporting information. A: Sholl Descriptors: Definitions, B: Sholl Descriptors: Constructions, C: Clustering Methods, D: Detection and Feature Selection, E: Metric Learning and Supervised Classification, F: Stability. Fig A: TMD as a Sholl-type descriptor. (a) Example of TMD-path decomposition on a simple planar tree. The soma marked with 1 is the root. Equicentered circles reveal the distances of nodes from the root. The furthest node is node 8. The paths from the TMD-path decomposition are: {[5, 4, 2], [3, 2, 1], [8, 6, 1], [7, 6]}, (b), The tree *T* with a single path *x* starting at the root *R*. When using TMD as a Sholl-type descriptor by considering TMD of *T* ∩ *B*(*R*, *r*) we will only see the final barcode [0, *d*(*R*, 6)] for *r* ≥ *d*(*R*, 4). For the radii *r* between *d*(*R*, 6) and *d*(*R*, 4) the endpoint of the persistence interval will be equal *r*. When *r* reaches *d*(*R*, 4) the endpoint of the persistence interval it will then jump down to *d*(*R*, 6). Fig B: Dendrogram of a bipolar interneuron. A representative bipolar cell is shown in (a). The “energy angle matrix” is used to separate the nodes into two clusters as shown in (b). The dendrogram can be read as follows: the angles between pairwise energy vectors associated to red nodes are small as is for the blue nodes. However, the angles between energy vectors of blue and red nodes are much larger. Fig C: Detection rate method. Method used to determine detection rate. Each circle is the boundary of a disk in the Euclidean metric. Fig D: Representation of isomorphic trees. Representative isomorphic trees with entirely different (a) branching pattern and (b) tortuosity. Fig E: Representative trees in the Hausdorff metric. Representative tree (a) and similar trees (b) and (c) that are close to tree (a) in the Hausdorff metric. Fig F: Instability. Instability behavior for tortuosity descriptor.
(PDF)
