# Supplementary figures and images for: Topological Sholl descriptors for neuronal clustering and classification
Source: PLoS Comput Biol. 2022 Jun 22;18(6):e1010229. doi: 10.1371/journal.pcbi.1010229 (PMC9255741; doi:10.1371/journal.pcbi.1010229)

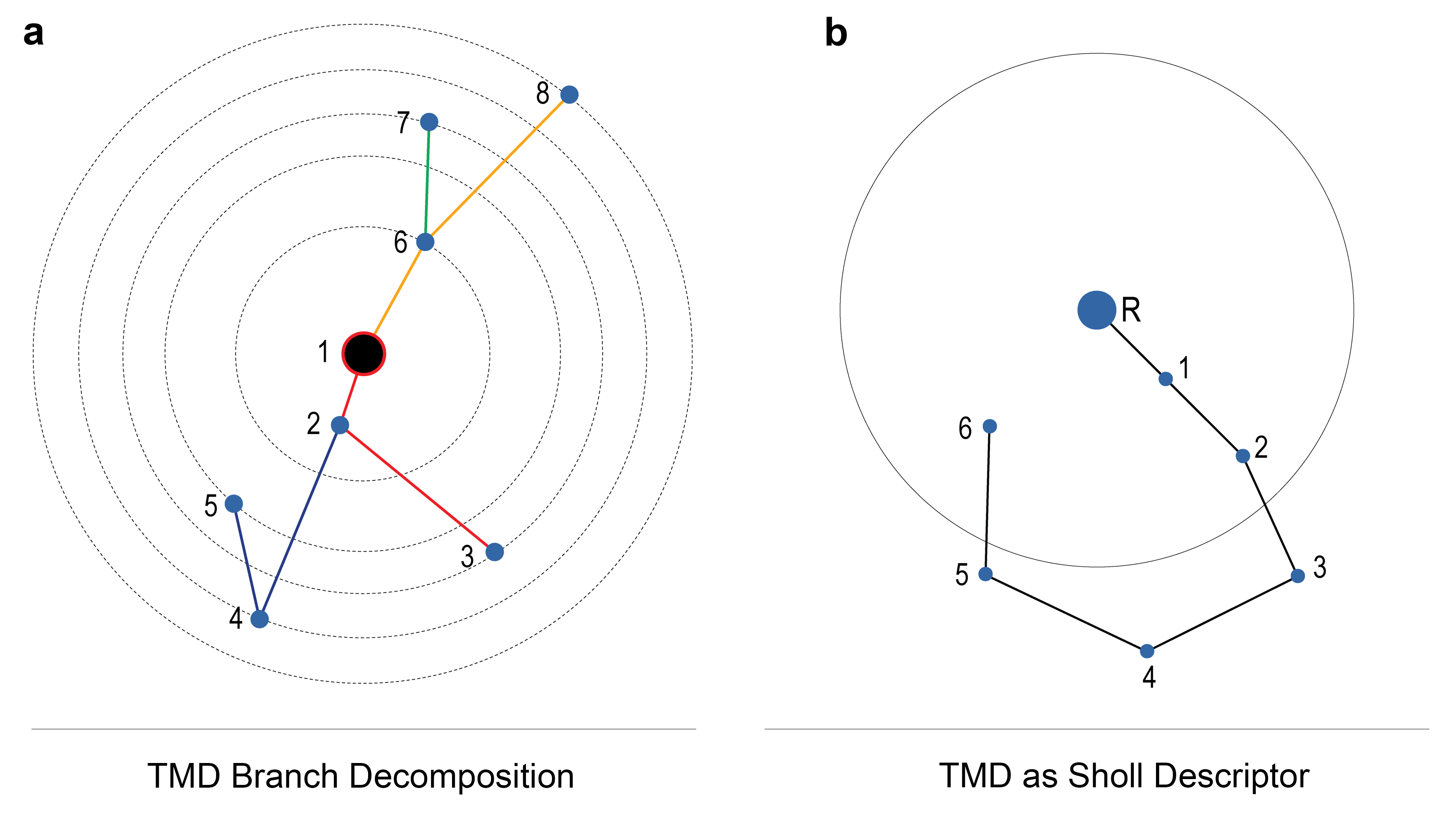

Supplement: S1 Fig — (a) Example of TMD-path decomposition on a simple planar tree. The soma marked with 1 is the root. Equicentered circles reveal the distances of nodes from the root. The furthest node is node 8. The paths from the TMD-path decomposition are: {[5, 4, 2], [3, 2, 1], [8, 6, 1], [7, 6]} (b), The tree T with a single path x starting at the root R. When using TMD as a Sholl-type descriptor by considering TMD of T ∩ B(R, r) we will only see the final barcode [0, d(R, 6)] for r ≥ d(R, 4). For the radii r between d(R, 6) and d(R, 4) the endpoint of the persistence interval will be equal r. When r reaches d(R, 4) the endpoint of the persistence interval it will then jump down to d(R, 6). (TIF) [file pcbi.1010229.s001.tif]

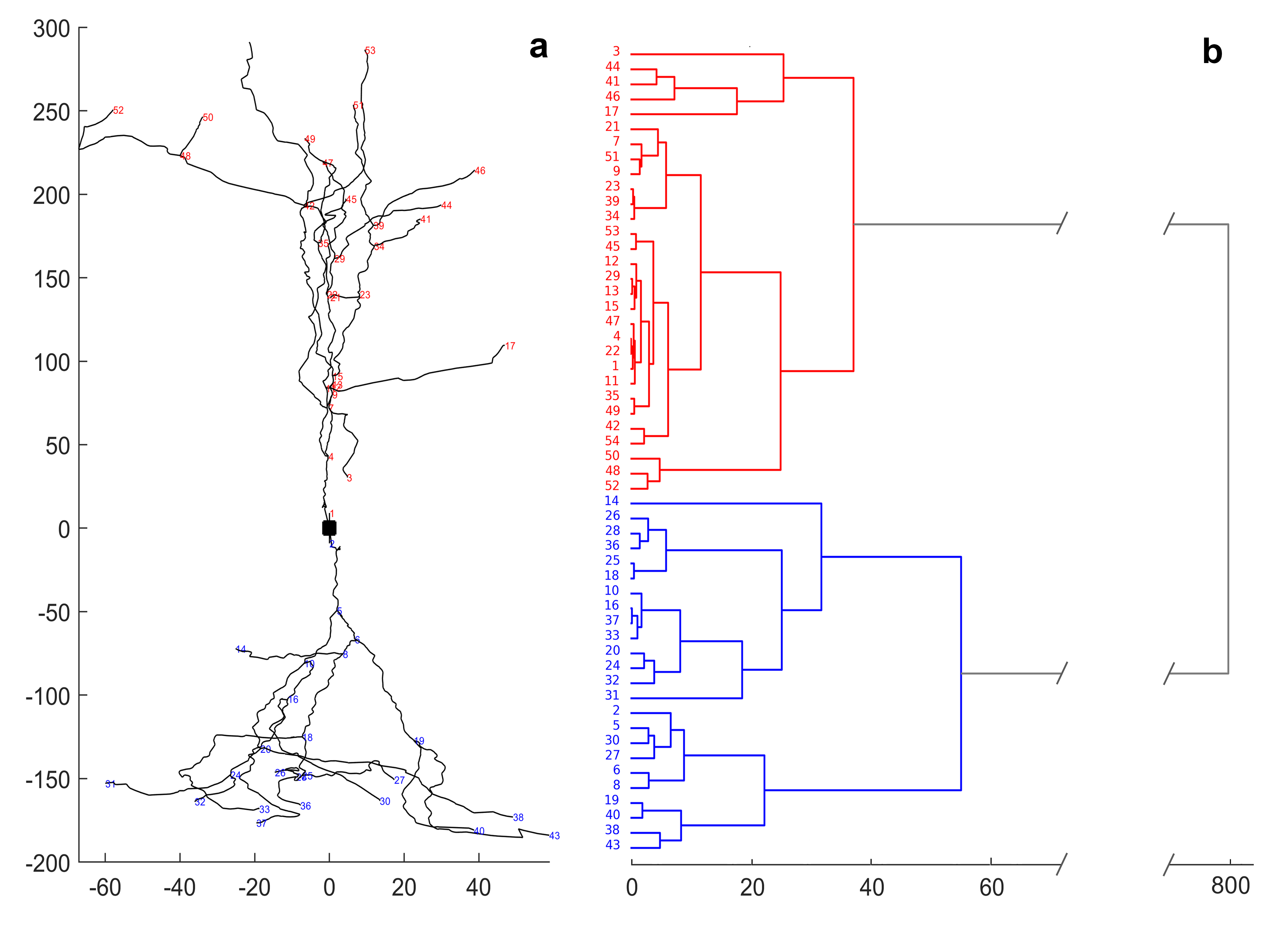

Supplement: S2 Fig — A representative bipolar cell is shown in (a). The “energy angle matrix” is used to separate the nodes into two clusters as shown in (b). The dendrogram can be read as follows: the angles between pairwise energy vectors associated to red nodes are small as is for the blue nodes. However, the angles between energy vectors of blue and red nodes are much larger. (TIF) [file pcbi.1010229.s002.tif]

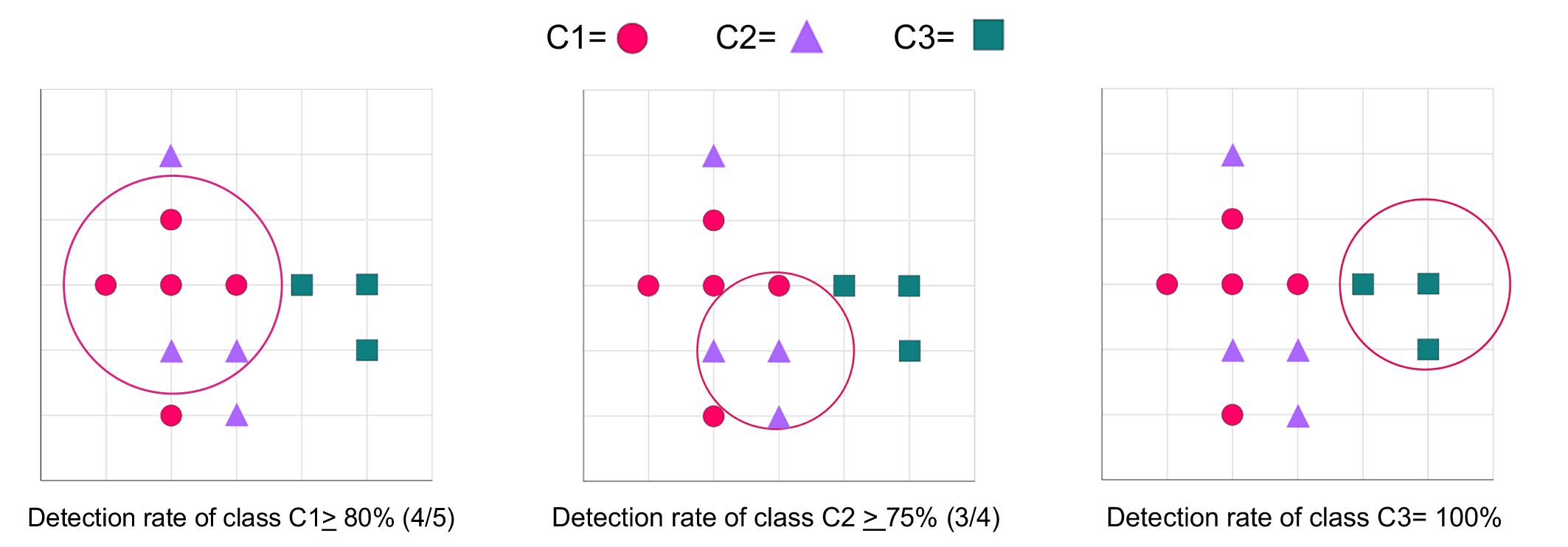

Supplement: S3 Fig — Method used to determine detection rate. Each circle is the boundary of a disk in the Euclidean metric. (TIF) [file pcbi.1010229.s003.tif]

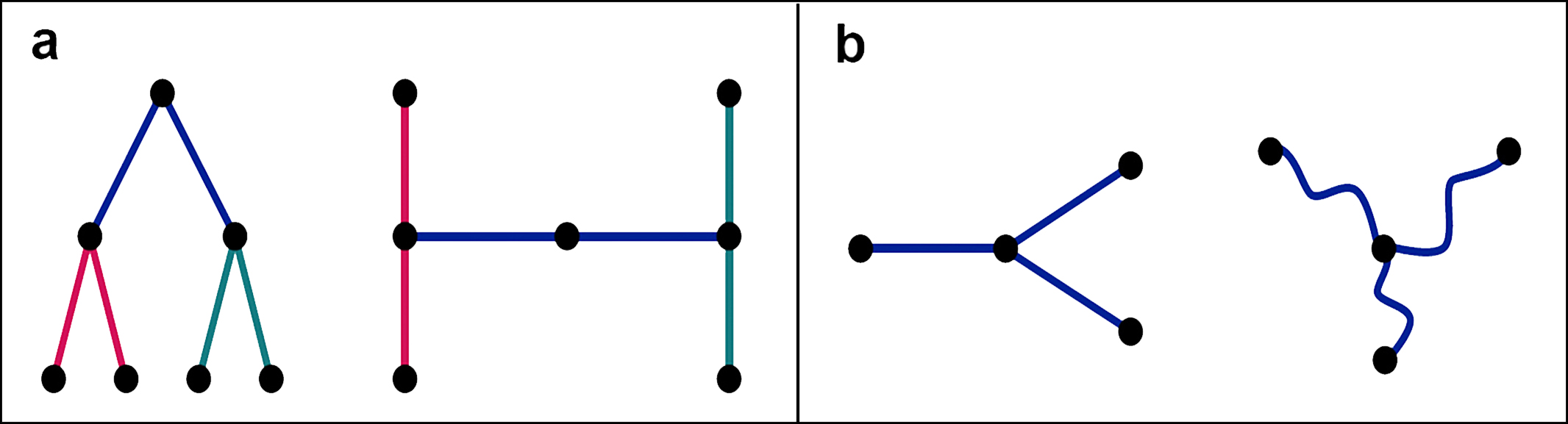

Supplement: S4 Fig — Representative isomorphic trees with entirely different (a) branching pattern and (b) tortuosity. (TIF) [file pcbi.1010229.s004.tif]

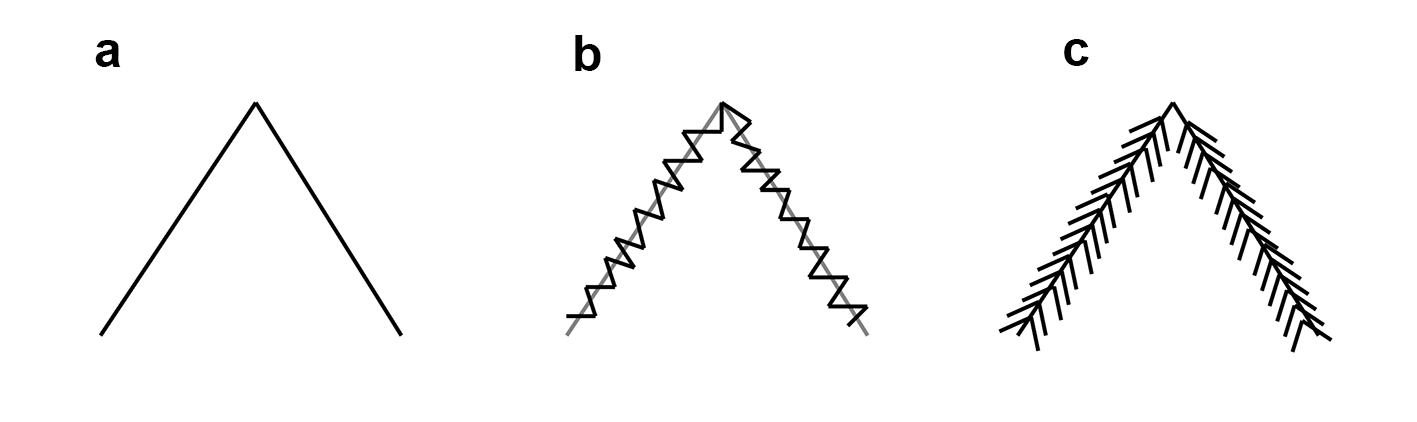

Supplement: S5 Fig — Representative tree (a) and similar trees (b) and (c) that are close to tree (a) in the Hausdorff metric. (TIF) [file pcbi.1010229.s005.tif]

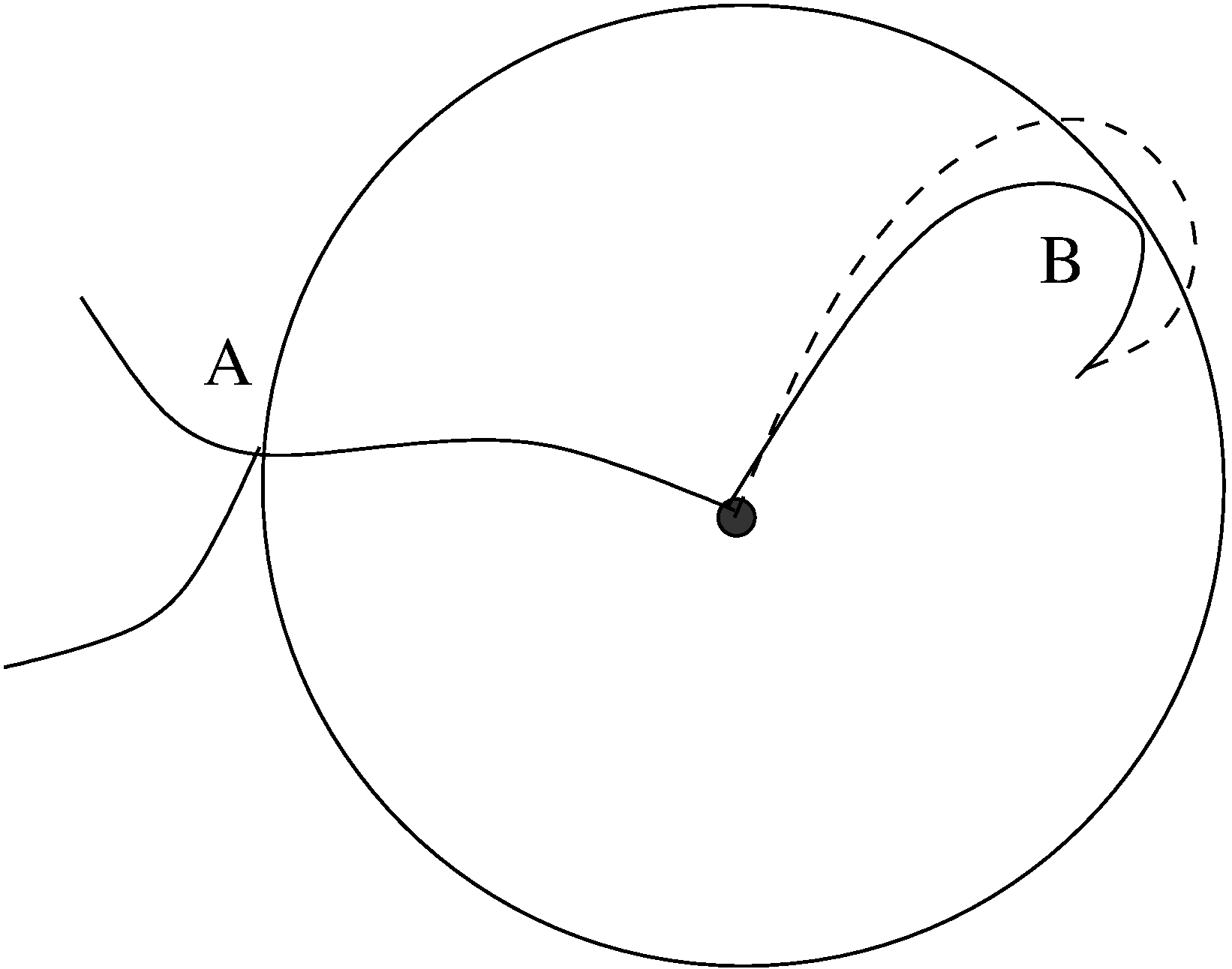

Supplement: S6 Fig — Instability behavior for tortuosity descriptor. (TIF) [file pcbi.1010229.s006.tif]
