# Supplementary material for: Topological Sholl descriptors for neuronal clustering and classification
Source: PLoS Comput Biol. 2022 Jun 22;18(6):e1010229. doi: 10.1371/journal.pcbi.1010229 (PMC9255741; doi:10.1371/journal.pcbi.1010229)
Supplement: S1 Text — A: Sholl Descriptors: Definitions, B: Sholl Descriptors: Constructions, C: Clustering Methods, D: Detection and Feature Selection, E: Metric Learning and Supervised Classification, F: Stability. Fig A: TMD as a Sholl-type descriptor. (a) Example of TMD-path decomposition on a simple planar tree. The soma marked with 1 is the root. Equicentered circles reveal the distances of nodes from the root. The furthest node is node 8. The paths from the TMD-path decomposition are: {[5, 4, 2], [3, 2, 1], [8, 6, 1], [7, 6]}, (b), The tree T with a single path x starting at the root R. When using TMD as a Sholl-type descriptor by considering TMD of T ∩ B(R, r) we will only see the final barcode [0, d(R, 6)] for r ≥ d(R, 4). For the radii r between d(R, 6) and d(R, 4) the endpoint of the persistence interval will be equal r. When r reaches d(R, 4) the endpoint of the persistence interval it will then jump down to d(R, 6). Fig B: Dendrogram of a bipolar interneuron. A representative bipolar cell is shown in (a). The “energy angle matrix” is used to separate the nodes into two clusters as shown in (b). The dendrogram can be read as follows: the angles between pairwise energy vectors associated to red nodes are small as is for the blue nodes. However, the angles between energy vectors of blue and red nodes are much larger. Fig C: Detection rate method. Method used to determine detection rate. Each circle is the boundary of a disk in the Euclidean metric. Fig D: Representation of isomorphic trees. Representative isomorphic trees with entirely different (a) branching pattern and (b) tortuosity. Fig E: Representative trees in the Hausdorff metric. Representative tree (a) and similar trees (b) and (c) that are close to tree (a) in the Hausdorff metric. Fig F: Instability. Instability behavior for tortuosity descriptor. (PDF) [file pcbi.1010229.s007.pdf]

TOPOLOGICAL SHOLL DESCRIPTORS FOR NEURONAL CLUSTERING AND CLASSIFICATION

Reem Khalil [1,*], Sadok Kallel [2], Ahmad Farhat [3], Pawel Dlotko [3]

*rkhalil@aus.edu

[1]American University of Sharjah, Department of Biology Chemistry and Environmental Sciences, Sharjah, United Arab Emirates.
[2]American University of Sharjah, Department of Mathematics, Sharjah, United Arab Emirates.
[3]Dioscuri Centre in Topological Data Analysis, Mathematical Institute, Polish Academy of Sciences, Warsaw, Poland.

Supplementary Material

Sholl descriptors are designed to analyze and differentiate neuronal morphology. The descriptors are combined with standard hierarchical clustering methods, a detection algorithm (also used for feature selection), grid search, and a metric learning algorithm are then used to cluster and classify datasets of neurons.

A. Sholl Descriptors: Definitions

As defined in §4.2, a Sholl descriptor is any rule Φ that associates to a given neuron $N \subset \mathbb{R}^3$ a compactly supported function $\tilde{\phi}_N$ whose independent variable is either path or radial distance from the soma, and whose values are in a metric space X. To each neuron and each feature ϕ, we write this associated Sholl function as $\tilde{\phi}_N : I \to X$, where I is either the interval $[0, R(N)]$ or $[0, L(N)]$. We further require this function to be both isometry invariant and stable with respect to reconstruction errors.

A.1. **Normalized Sholl Functions.** We normalize the Sholl function $\tilde{\phi}$ so that the resulting function is supported on $[0, 1]$. Suppose $\tilde{\phi}_N$ is supported on $[0, R(N)]$ for example, where $R(N)$ is the span of the neuron. Then we define the corresponding normalized function ϕ_N by:

$$\phi_N : [0,1] \longrightarrow X$$
$$\phi_N(r) = \tilde{\phi}_N(rR(N))$$

If $\tilde{\phi}_N$ is a function of path length, and thus supported on $[0, L(N)]$, then we normalize in the same way, with $L(N)$ replacing $R(N)$. If $\tilde{\phi}$ is isometry invariant to begin with, then so is ϕ. Being isometry invariant means that for any linear transformation A of $\mathbb{R}^3$, $\phi_{AN}(r) = \phi_N(r)$, where AN is the image of $N \subset \mathbb{R}^3$ by the transformation A.

Remark A.1. In this paper, all our descriptors will be normalized and supported on $[0, 1]$. The constructions we provide are such that all real-valued Sholl functions we consider are step functions.

A.2. **Sholl Descriptors and Functional Metrics.** Given a morphological feature ϕ and a family $\mathcal{N}$ of neurons, the association

$$\Phi : \mathcal{N} \longrightarrow \mathrm{Map}(I, X) \quad , \quad N \longmapsto \phi_N \tag{1}$$

where ϕ_N is the Sholl function discussed above, is called a *Sholl descriptor*. If $X = \mathbb{R}$, Sholl descriptors form an algebra, in particular they form a vector space over $\mathbb{R}$. Indeed, given two normalized Sholl functions $\phi_1, \phi_2 : I \to \mathbb{R}$, we can take linear combinations. This sum is also stable, as defined in §F in S1 Text, if we start with stable descriptors.

Each Sholl descriptor Φ defines a pseudo-metric on $\mathcal{N}$ as follows. Let d be any metric on the space of functions $\mathrm{Map}(I,X)$. It induces a pseudo-metric d_ϕ on $\mathcal{N}$ by setting

$$d_\phi(N_1,N_2) := d(\phi_{N_1},\phi_{N_2}) \quad , \quad \text{for all } N_1,N_2\in\mathcal{N} \tag{2}$$

Note that, since ϕ is $O(3)$-equivariant, we have that $d_\phi(N_1,N_2)=0$ if N_1 is the image of N_2 by a rotation or a reflection.

The existence of the pseudo-metrics allows us to conclude that: the more similar N_1 and N_2 are in the feature ϕ, the smaller is $d_\phi(N_1,N_2)$. This statement is at the basis of our stability results (§F in S1 Text).

The functional distance d on the space $\mathrm{Map}(I,X)$ we choose to work with is the "L^1 distance"

$$d(\phi_{N_1},\phi_{N_2}) := \int_0^1 d_X(\phi_{N_1}(r),\phi_{N_2}(r))dr \tag{3}$$

All ϕ_N constructed in this paper are real-valued *step functions*, except for the Sholl-TMD in §B.7 in S1 Text . We give the formula for the distance in this case. Let ϕ_1,ϕ_2 be two step functions with jumps at radii $r_1,\dots,r_q$ and $s_1,\dots,s_\ell$ respectively. This means that ϕ_1 is constant on $[r_i,r_{i+1}[$, and similarly ϕ_2 is constant on $[s_j,s_{j+1}[$. Let

$$\{t_1,\dots,t_{q+\ell}\} = \{r_1,\dots,r_q\}\cup\{s_1,\dots,s_\ell\} \tag{4}$$

and order the t_i's by increasing magnitude so we can assume

$$0=t_0<t_1<t_2\le\cdots<t_{q+\ell} \tag{5}$$

Then the L^1-distance between the step functions is given by

$$d(\phi_{N_1},\phi_{N_2}) := \int_0^1 |\phi_{N_1}(r)-\phi_{N_2}(r)|dr = \sum_{i=0}^{q+l-1}(t_{i+1}-t_i)|\phi_{N_1}(t_i)-\phi_{N_2}(t_i)| \tag{6}$$

This distance is now set to be equal to $d_\phi(N_1,N_2)$.

Remark A.2. Other choices of metrics we can work with for real valued Sholl functions are the L^p metrics for $p>1$ or the Sup metric $d(\phi_{N_1},\phi_{N_2}) := \mathrm{Sup}_{r\in[0,1]}(\phi_{N_1}(r);\phi_{N_2}(r))$. The first one doesn't differ much from L^1, and the second is very weak in detecting real changes, and thus is not suitable. In future work, we will introduce other metrics, and will investigate their effects and stability properties. Nevertheless, once we can measure functional distances between descriptor functions, we can measure "Sholl distances" between neurons as indicated in Eq.(2) in S1 Text and use them to cluster and classify neurons.

B. Sholl Descriptors: Constructions

Given a neuron N viewed as an embedded tree in space, we define

$$N_r := (N\cap B_r)_c = \text{connected component of } N\cap B_r \text{ containing the soma} \tag{7}$$

Here B_r is the ball of radius r around the soma.

B.1. **The Branching Pattern Descriptor.**

This morphological descriptor detects patterns that results from the distribution of branches and leaves relative to the soma (see Fig 3).

Let N be a neuron which we view as a collection of single rooted binary trees in $\mathbb{R}^3$, with the common root being the Soma. Label $B_1,\dots,B_q$ the branch points of N and label all leaves by $L_1,\dots,L_k$. Let $r>0$ be the radial distance measured away from the Soma. Order the branch points and leaves by increasing r, so that if r_i indicates the distance of the i-th node to the soma, we have $0<r_1<r_2<\cdots<r_k$ (equal radii can be removed by an infinitesimal perturbation).

Fixing a neuron N as before, associate to each $r\in I$ the number $\alpha(r)$ defined by

$$\alpha(r) = \#\{B_i\ | r_i\le r\} - \#\{L_j\ |\ r_j\le r\} \tag{8}$$

Let $R(N)$ be the span of the neuron N and define the function

$$\tilde{\phi}_N : [0,R(N)]\longrightarrow\mathbb{R}^+\ ,\ r\longmapsto\alpha(r) \tag{9}$$

This is a step function since $\alpha(r)$ is constant on intervals $r_i \leq r < r_{i+1}$. The normalized version takes the form

$$\phi_N : [0,1] \longrightarrow \mathbb{R}^+ \ , \ \phi_N(r) = \tilde{\phi}_N(rR(N)) \tag{10}$$

This defines our branching-pattern descriptor ϕ. This descriptor is isometry invariant (it only depends on the distance of the branchpoints from the soma) and so it is well-defined as a function on the isometry classes of neurons as already indicated. Note that

$$\phi_N(1) = \#\text{all branch points} - \#\text{number of all leaves} = -\#\text{primary dendrites of N} \tag{11}$$

since the number of primary branches is the difference between the number of leaves and the number of bifurcation points.

Algorithm Branching

Input: Tree T with a root r
Let $N = \{n_1, \ldots, n_k\}$ be a vector of branching points and leafs in T sorted by their distance to r
$number\_of\_branches = 0$, $R = []$
for $i = 1$ to k **do**
    if n_i is a branching node **then**
        $number\_of\_branches = number\_of\_branches + 1$
    else
        $number\_of\_branches = number\_of\_branches - 1$
    end if
    $R = R \cup [d(n_i, r), number\_of\_branches]$
end for
return R

B.1.1. *Tortuosity Descriptor.* Given a representation of a neuron N, let us label its nodes by $P_1, \ldots, P_n \in \mathbb{R}^3$. For any two nodes, we can consider both path distance and Euclidean distance between them. If P_i is a parent node and P_j is the child node, let $b_{i,j}$ be the dendritic path distance between these nodes, and let $d_{i,j}$ be the length of the segment $[P_i, P_j]$. The ratio of both distances is

$$\delta_{i,j} = \frac{b_{i,j}}{d_{i,j}} \quad \text{tortuosity of a branch between } P_i \text{ and } P_j \tag{12}$$

For a neuron N with n nodes §4.1 there are exactly $n-1$ such (parent,child) pairs. Define the average tortuosity of N to be the average

$$\alpha(N) = \frac{1}{n-1}\sum_{(i,j)} \delta_{i,j} \quad , \quad (i,j) \text{ running over all (parent-child) pairs} \tag{13}$$

It is clear that $1 \leq \alpha(N)$ for all choices of N. The (non-normalized) Sholl descriptor function associated to this construction is now given as follows: order the the nodes of N by increasing radii as before. Then define

$$\begin{aligned} T_N : [0, R(N)] &\longrightarrow \mathbb{R} \\ r &\longmapsto \alpha(N_r) \end{aligned}$$

We then take the normalized version §A.1 in S1 Text.

Algorithm Tortuosity

Input: A tree T with a root r
Let $P_1, P_2, \ldots, P_n$ be branching and termination nodes of T sorted by their distance to r
$R = []$
for $n = 1 \to n$ **do**
    Compute $N_{d(P_i,r)}$
    $T_i = \alpha(N_{d(P_i,r)})$
    $R = R \cup [d(P_i, r), T_i]$
end for
return R

B.2. **Taper Rate Descriptor.** We start with a neuron N and list all *path distances* of the nodes to the soma in increasing order $0 < \ell_1 < \cdots < \ell_k$. Each node has a dendritic thickness (or width) that tapers as we move away from the soma along the dendrite. We can measure the tapering rate as a function of path distance. More precisely, define

$$TP_N(r) = \text{dendrite thickness at the node at } \ell_i, r \in [\ell_i, \ell_{i+1}[\tag{14}$$

and then take the associated normalized Sholl descriptor by dividing ℓ_i by the length of the longest dendrite. This is a Sholl function whose variable is path length and not radial distance.

B.3. **Flux Descriptor.** Given a neuron N, we define the flux F and the associated normalized flux function $F_N : [0,1] \longrightarrow \mathbb{R}$. For $r > 0$ and a dendrite crossing the boundary sphere S_r at a point $P \in N \cap S_r$, we identify the parent and the child of N. Assuming they are on the either side of the sphere, the direction vector $\overrightarrow{AB}$ from parent to child points outward if A is inside, and points inward if A is outside the sphere. Consider the segment $[A, B]$ and let C be the point on the intersection of the segment and the sphere. We then assign the value

$$f_N(P) = \frac{\overrightarrow{AB}}{|AB|} \cdot \frac{\overrightarrow{OC}}{|OC|} \tag{15}$$

where O is the root of the tree. This is the cosine of the angle between the unit vector along $\overrightarrow{AB}$ and the normal to the sphere going through C. This value is maximal if $\overrightarrow{AB}$ is aligned with the radial vector at C, so the angle is zero.

To define the total flux function, order the nodes of N as before by increasing values of their distances from the soma $0 < r_1 < \ldots < r_k$. For every $r = \dfrac{r_i + r_{i+1}}{2}$, take the sphere of that radius r, look at all dendrites intersecting that sphere at $P_1, \ldots, P_k$ and add up the values obtained from the construction outlined above. This value is

$$F_N(r) = \sum_i f_N(P_j) \quad , \quad P_j \in N \cap S_r \tag{16}$$

This gives rise again to a step function $F_N : [0,1] \longrightarrow \mathbb{R}$ by setting

$$F_N(r) := F_N\left(\frac{r_i + r_{i+1}}{2}\right) \text{ if } r \in \left[\frac{r_i + r_{i+1}}{2}, \frac{r_{i+1} + r_{i+2}}{2}\right[\tag{17}$$

Additionally, if $A = (a_1, a_2, a_3)$ is the parent marker and $B = (b_1, b_2, b_3)$ the child marker, such that either $|OA| < r < |OB|$ or $|OB| < r < |OA|$, that is on different sides of the sphere, the point of intersection $C = (c_1, c_2, c_3)$ of that sphere with the segment $[A, B]$ is obtained by setting $r^2 = c_1^2 + c_2^2 + c_3^2$ and $C = (1-t)A + tB$, then solving for t through a quadratic. The flux value at C is

$$f_N(C) = \frac{c_1(b_1 - a_1) + c_2(b_2 - a_2) + c_3(b_3 - a_3)}{\sqrt{(c_1^2 + c_2^2 + c_3^2)((b_1 - a_1)^2 + (b_2 - a_2)^2 + (b_3 - a_3)^2)}} \tag{18}$$

Algorithm Flux

```
Input: Tree T with a root r
For every branch e_i ∈ T let r_i be the distance of the mid-point of e_i to r.
Let [r_1, r_2, ..., r_k] be the sorted vector of those distances.
F = []
for i = 1 to k do
    S_i = sphere of a radius r_i centered in r.
    For an edge e ∈ T s.t. S_i ∩ e ≠ ∅, let v_{i,e} be a vector orthogonal to S_i starting in S_i ∩ e.
    f_i = Σ_{e∈T | e∩S_i≠∅} cos(∠(v_{i,e}, e))
    F = F ∪ [r_i, f_i]
end for
return F
```

B.4. **The Leaf Index Descriptor.** The leaf index of a node P, denoted as $\mathrm{li}(P)$, is the the number of leaves that can be reached from P along paths that go away from the soma. When P is a leaf, we set $\mathrm{li}(P) = 1$.

To compute a leaf index of a neuron N, order its nodes $P_1, \ldots, P_q$ by increasing order of path distance to the soma $0 < r_1 < \cdots < r_k$, and define the Leaf Index Sholl Descriptor as follows:

$$LI_N : [0,1] \longrightarrow \mathbb{N} \quad , \quad r \longmapsto \begin{cases} \mathrm{li}(rL(N)) & \text{if } r \in [r_i, r_{i+1}[\\ 1 & r = 1 \end{cases}$$

Evidently $LI_N(1) = 1$, which is the value at the furthest leaf, while $LI_N(0)$ is the total number of leaves. This is again a step function and distances between leaf index Sholl functions can be given by the standard formula Eq.(6) in S1 Text.

Algorithm Leaf Index

1. Input: A tree T with a root r
2. Let $[r_1, r_2, \ldots, r_k]$ be a sorted vector of distances between branching and termination nodes $n_1, n_2, \ldots, n_k$ of T and r
3. $N_i =$ the number of leaves having n_i as a predecessor
4. **return** $[(r_1, N_1), (r_2, N_2), \ldots (r_k, N_k)]$

B.5. **Total Wiring Descriptor.** "Total wiring" is a morphological feature which measures the total dendritic length of neurons. When used as a Sholl descriptor, it gives total length of dendrites, but also their density as we move away from the soma.

Given a neuron N, let $t\ell(N)$ be the total length of all dendrites of N. Let N_r be the connected component of N that contain the soma within a sphere of radius r centered at the soma Eq.(7) in S1 Text. Then

$$TL_N : [0,1] \longrightarrow \mathbb{R}^+ \tag{19}$$

$$r \longmapsto t\ell(N_{rR(N)}) \tag{20}$$

This is a normalized Sholl function, which always starts at value 0 and ends up at value $TL_N(1) = t\ell(N)$ which is the total wiring of the neuron. As for other Sholl functions, we will only consider the step function version of this construction, where once more one defines for $r \in [0,1]$,

$$TL_N(r) = \begin{cases} t\ell(N_{r_i R(N)}) & \text{if } r \in [r_i, r_{i+1}[\\ t\ell(N_{r_k R(N)}) & \text{if } r \in [r_k, 1] \end{cases} \tag{21}$$

where $0 < r_1 < \ldots < r_k \leq 1$ are the normalized radial distances of the nodes listed in increasing order.

Algorithm Wiring

1. Input: A tree T with a root r
2. Let $n_1, n_2, \ldots, n_k$ be branching and termination nodes of T sorted by their distance to r
3. $D_0 = 0$, $R = []$
4. **for** $i = 1 \rightarrow k$ **do**
5. $\quad d =$ length of path from n_i to nearest branching node in direction of r.
6. $\quad D_i = D_{i-1} + d$
7. $\quad R = R \cup [d(n_i, r), D_i]$
8. **end for**
9. **return** R

B.6. **Energy Descriptor (Nodal Distribution).** Given a neuron N, consider all its nodes as a point cloud in 3D space containing one charged particle per point. The charge each node carries will be proportional to the thickness of the branch at that point, if available and 1 otherwise. These charged nodes affect the space around them through the electric field they generate. This electric field is a well-defined map

$$E_N : \mathbb{R}^3 \setminus \{\text{nodes}\} \longrightarrow \mathbb{R}^3 \tag{22}$$

Taking the intensity of the vector field at each point of $\mathbb{R}^3 \setminus \{\text{nodes}\}$ gives us a measure of how space is being affected by the neuron. This also gives a measure of how the nodes are distributed in space as we will later illustrate in the case of Purkinje cells.

Let $\zeta := \{P_1, \dots, P_n\}$ be the nodes of N, with P_i having charge q_i. Each point $P_i = (x_i, y_i, z_i)$ of ζ contributes an "electric vector field" of the form

$$E_i(x,y,z) = q_i \left(\frac{x - x_i}{|PP_i|}, \frac{y - y_i}{|PP_i|}, \frac{z - z_i}{|PP_i|} \right) \tag{23}$$

where $|PP_i| = \sqrt{(x-x_i)^2 + (y-y_i)^2 + (z-z_i)^2}$. By superposition, all nodes together $\zeta = \{P_1, \dots, P_n\}$ generate a vector field $E_N(x,y,z) = \sum_i q_i F_i(x,y,z)$ with square intensity

$$|E_N(x,y,z)| = \left(\sum q_i \frac{x - x_i}{|PP_i|}\right)^2 + \left(\sum q_i \frac{y - y_i}{|PP_i|}\right)^2 + \left(\sum q_i \frac{z - z_i}{|PP_i|}\right)^2$$

Let $O(2)$ be the group of orthogonal matrices. This group acts on $\mathbb{R}^3$, and thus on the set of neurons.

Lemma B.1. *The intensity at the soma, $|E_N(0,0,0)|$, is $0(2)$-invariant.*

Proof. If $A \in O(2)$ and $N \subset \mathbb{R}^3$ a neuron, we write $A(N)$ the image of N under this action. We argue first that $|E_{A(N)}(P)| = |E_N(A^{-1}(P))|$ for $P \in \mathbb{R}^3$. The nodes of N are $\{P_1, \dots, P_n\}$. Write $E_{A(N)}(P) = \sum E_{A(P_i)}(P)$. Since $E_{A(P_i)}(P) = AE_{P_i}(A^{-1}(P))$, and since A is linear and preserves lengths, it follows that

$$|E_{A(N)}(P)|^2 = |\sum E_{A(P_i)}(P)| = |\sum AE_{P_i}(A^{-1}(P))| = |A \sum E_{P_i}(A^{-1}(P))| \tag{24}$$

$$= |\sum E_{P_i}(A^{-1}(P))| = |E_N(A^{-1}(P)| \tag{25}$$

At the soma $P = (0,0,0)$, $A^{-1}(P) = P$, so that $|E_{A(N)}(P)| = |E_N(P)|$, which is what is claimed. ☐

Our Sholl descriptor associates to every neuron the map

$$\phi_N : [0,1] \longrightarrow \mathbb{R}^+ \tag{26}$$

$$r \longmapsto |E_{N_r}(0,0,0)| \tag{27}$$

where N_r is as in Eq.(7) in S1 Text. This map adds the unit vectors at the soma, one for each node, and takes the magnitude. We can also think of energy as the effect of the nodal distribution around the soma. If all nodes are on one side of a plane going through the soma, then their contributions is greatest (eg. Purkinje cells have very large energy values), as opposed to nodes that are evenly distributed around the soma. In this latter case, several cancellations occur and the energy value tends to be small.

Algorithm Energy

Input: Tree T with a root r and a function $diam : T \to \mathbb{R}$ describing diameter of T.
Let N be the set of all branching nodes and leafs in T
For every $v_i \in N$, let r_i be the Euclidean distance between v_i and r
Let $[r_1, r_2, \dots, r_k]$ be a sorted vector of distances.
for $n = 1 \to k$ **do**
    v_i is a vector in the direction of a line between r and n_i of a length equal to $diam(n_i)$.
end for
$E_0 = 0$, $d_0 = [0,0,0]$, $R = []$
for $n = 1 \to k$ **do**
    $d_i = d_{i-1} + v_i$
    $E_i = |d_i|$
    $R = R \cup [r_i, E_i]$
end for
return R

Remark B.2. (Polarization) The Sholl Energy descriptor can be utilized effectively in determining if a given neuron is either polarized or not. The observation here is that a neuron is *polarized* if the pairwise angles between energy vectors contributed by the various nodes all lie in a relatively small range. A bipolar

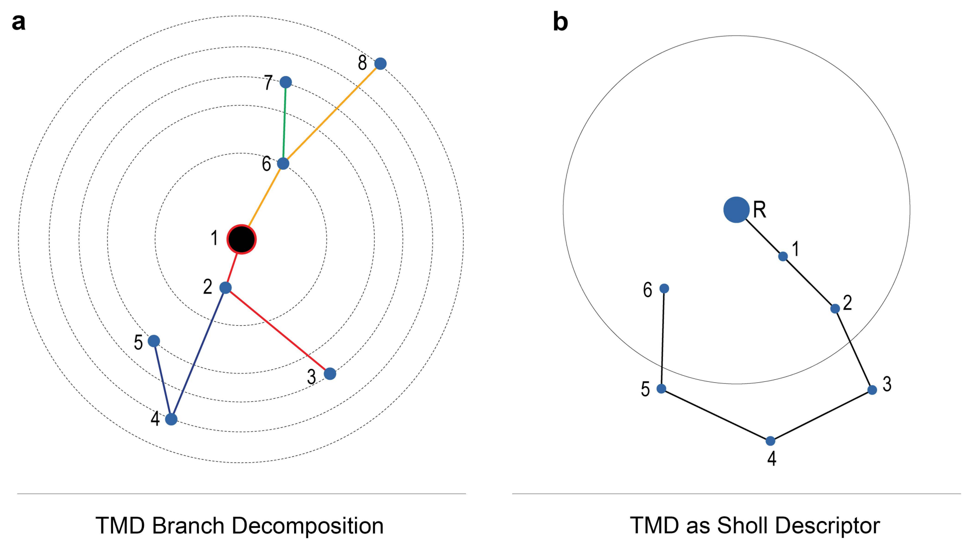

Fig A –TMD as a Sholl-type descriptor. (**a**) Example of TMD-path decomposition on a simple planar tree. The soma marked with 1 is the root. Equicentered circles reveal the distances of nodes from the root. The furthest node is node 8. The paths from the TMD-path decomposition are: $\{[5,4,2],[3,2,1],[8,6,1],[7,6]\}$ (**b**), The tree T with a single path x starting at the root R. When using TMD as a Sholl-type descriptor by considering TMD of $T \cap B(R,r)$ we will only see the final barcode $[0, d(R,6)]$ for $r \geq d(R,4)$. For the radii r between $d(R,6)$ and $d(R,4)$ the endpoint of the persistence interval will be equal r. When r reaches $d(R,4)$ the endpoint of the persistence interval it will then jump down to $d(R,6)$.

cell always produces two distinct clusters of vectors such that angles between vectors within a cluster are small, and angles between vectors in different clusters are large. This is explained in more details in §C.3 in S1 Text.

B.7. **The Topological Morphological Descriptor (TMD).** Let $T \subset \mathbb{R}^3$ be a tree with a root R. A *path* is any continuous sequence of edges in T. Each path x have unique initial $b(x)$ and terminal $d(x)$ vertices. The TMD is based on a method of decomposing a given tree T into a collection of paths such that the sum of those paths is the whole tree T. In addition, any two paths from that decomposition will either have an empty intersection, or their intersection is the endpoint of one of them (and in this case a branchpoint of T).

The TMD path decomposition is obtained using the following procedure; All the paths from the TMD-path decomposition starts at the leaves of T. They continue along the tree, towards the root, until they reach a node n of degree 3 or higher in T. In the node n all the paths except from one terminate. The path that continues through the node n is the one with the initial node further away from R (soma)[1]. Once a path reaches the root R, it does not continue any further (it terminates). For example, a TMD-path decomposition is presented in Fig A in S1 Text.

Given a TMD-path decomposition as described above, a collection of pairs of numbers inspired by *persistent diagram* is associated to this decomposition. For that purpose, a path x having the initial and terminal vertices $b(x)$ and $d(x)$ correspond to persistence interval $[d(R, b(x)), d(R, d(x))]$. Using the terminology from persistent homology, we say that the path x is *born* at the radius $d(R, b(x))$ and *dies* at the radius $d(R, d(x))$. The collection of all such birth-death pairs is then used as a signature of the tree T. As the obtained signature has the structure of persistent diagram, we further adopt various metrics from persistent homology to compare such diagrams.

To fit the TMD into the scheme of the current paper we will now turn it into a Sholl descriptor having values in the space of persistence diagrams. For that purpose let T_r be the connected component of $T \cap B(R,r)$ containing R. Let us make two simple observations:

[1]In case of two or more paths satisfying this condition, the one that continue is picked up randomly.

(1) Note that the TMD-path decomposition of T restricted to T_r is a valid TMD-path decomposition of T_r. To see that, let us consider a branching node n in T, such that $d(R, n) \leq r$, and the same node in T_r. The paths from n to the leaves in T_r will either be the same as in T, or they will be cut short in T_r by $B(R, r)$. In both cases, the path that does not terminate at n in T will also be the path with the initial point further away from R in T_r. It is possible that more than one path in T_r joining in n will be cut short by $B(R, r)$. However in this case we can choose to continue in T_r the same path that continues in T. Consequently, a TMD-path decomposition in T_r can be obtained by appropriate restriction of the TMD-path decomposition in T.

(2) Suppose we consider a path x in T giving rise to the persistence interval $[d(R, b(x)), d(R, d(x))]$. Then the path x will be present in T_r for $r \geq d(R, d(x))$. However it may happen that the path x contains points that are further away from R than $d(R, b(x))$ and will be cut in those points by $B(R, r)$. This will happen when the path x turns around as presented in the Fig A in S1 Text. In that instance, the interval $[d(R, b(x)), d(R, d(x))]$ in T_r for certain values of r will have a larger value of the first coordinate than the corresponding interval in T. Therefore, while the first (birth) coordinate of the interval corresponding to x in T_r may be longer than the interval corresponding to x in T. The actual length can be obtained from the coordinates of degree-2 vertices in x.

Those two observations allows for quick computation of the Sholl version of TMD descriptor, i.e. a TMD of a tree T_r, also denoted as $TMD(T, r)$. Firstly the TMD-path decomposition P of T is computed. Subsequently, for a given radius r, a subset $P' \subset P$ containing all the paths x such that $d(x) \leq r$ is selected. The paths in $x \in P'$ are transversed to find the point f_x in there which is inside $B(R, r)$ and furthest away from its center. Once found, the pair $(d(R, f_x), d(x))$ is added to the $TMD(T, r)$.

The algorithm described above uses the radial distance from the soma to construct T_r. When an intrinsic distance is used instead both in TMD and in construction of T_r, the Sholl version of the descriptor is even easier to obtain, as each path $x \in P$ such that $d(x) \leq r$ will give rise to a pair $(d(x), \min(b(x), r))$ in $TMD(T, r)$.

Unlike other Sholl descriptors, $TMD(T, r)$ has the range in the space of persistence diagrams which is a much richer mathematical structure than real numbers. Yet, it is still possible to compute distances between the functions $TMD(T, r)$ and $TMD(T', r)$. Let us assume that both functions has been computed for a discrete set of values $0 = r_0 < r_1 < r_2 < \ldots < r_n$. Then a distance between $TMD(T, r)$ and $TMD(T', r)$ can be approximated by:

$$\sum_{i=0}^{n-1} (r_i - r_{i-1})\ d_{diag}(TMD(T, r_i), TMD(T', r_i)) \tag{28}$$

where d_{diag} denotes any distance between persistence diagrams, e.g. p-Wasserstein distance.

Algorithm Sholl-TMD

```
Input: A tree T with a root r
Step 1: Find a branch decomposition of T
branches : T → T – for every node of T, returns the endpoint of a branch the node is in
L = the leafs in T
for l ∈ L do
    f(l) = l
end for
Branches = []
while L ≠ ∅ do
    M = ∅
    for n ∈ L do
        Let m be a predecessor of n in T
        if f(m) is not defined and all children of m have assigned value of f then
            k = children of m with largest value of d(f(k), r)
            f(m) = f(k)
            for k′, children of m, k′ ≠ k do
                Branches = Branches ∪ [f(k′), m]
            end for
            M = M ∪ m
        end if
    end for
    L = M
end while
Step 2: Compute Sholl-TMD
Let [r_1, r_2, ..., r_k] be branching points of T
FPD = []
for i = 1 → k do
    PD = []
    for [a, b] ∈ Branches s.t d(a, r) ≤ r_i do
        if Entire branch [a, b] ⊂ B(r, r_i) then
            PD = PD ∪ [d(a, r), d(b, r)]
        else
            if There exist a point c in between a and b s.t. d(c, r) = r_i then
                PD = PD ∪ [d(a, r), r_i]
            end if
        end if
    end for
    FPD = FPD ∪ [r_i, PD]
end for
```

C. Clustering Methods

Given a Sholl descriptor ϕ, and a family of neurons $N_1, \ldots, N_q$, we generate a distance matrix associated to the descriptor

$$[d_\phi(N_i, N_j)]_{1 \leq i,j \leq q}$$

This is a symmetric matrix with non-negative entries, and zeros along the diagonal. Any such distance matrix produces a dendrogram using standard hierarchical clustering algorithms. It is not reasonable to expect a single descriptor to cluster faithfully a given set of classes of neurons. The advantage of developing multiple descriptors based on various morphological features reveals which features are uniform and which are different among classes of neurons. By combining descriptors linearly §C.1 in S1 Text or via vectorization §C.2 in S1 Text, we can obtain better clustering.

C.1. **Optimal Combination of Metrics.** Let us assume that the considered collection of neurons belongs to a number of classes. We present a greedy grid–based search procedure that can reveal which features

are ‘fundamentally’ different between the classes. For that purpose, we consider a new distance d being the following linear combination:

$$d = \alpha_1 \; d_1 + \alpha_2 \; d_2 + \ldots + \alpha_n \; d_n,$$

where the non-negative constants α_i are sampled from a uniform grid.

Consider first the situation when each neuron may belong to one of two classes. For the fixed choice of $\alpha_1, \ldots, \alpha_n$ let $I_k = \max_{x,y \in C_k} d(x,y)$ be the maximal distance d between objects in C_k for $k \in \{1,2\}$. Let $E = \{\min_{x \in C_1, y \in C_2} d(x,y)\}$ be the minimal distance d between elements from two different classes. We then consider the ratio

$$sc_{\alpha_1,\ldots,\alpha_n} = \frac{E}{I_1 + I_2}$$

and select $\alpha_1, \ldots, \alpha_n$ from our grid of points that maximize $sc_{\alpha_1,\ldots,\alpha_n}$. Note that this greedy grid search has exponential complexity with n, being the number of considered distances.

The obtained weights $\alpha_1, \ldots, \alpha_n$ give an idea of the relative importance of different distances in the separation of classes C_1 and C_2. Consequently, when features leading to distances $d_1, \ldots, d_n$ can be interpreted geometrically, the weights may help identify the geometrical features that are important in the separation of the classes, and those that are not.

This idea can be generalized for a multi–class problem. There are various ways this can be achieved and we will present one possible approach. Let us have k classes $C_1, \ldots, C_k$ and define $I_j = \max_{x,y \in C_j} d(x,y)$ (maximal internal distance in C_j) and $E_{i,j} = \{\min_{x \in C_i, y \in C_j} d(x,y)\}$ (minimal distance between classess C_i and C_j). Then the multi-class score is given by:

$$sc_{\alpha_1,\ldots,\alpha_n} = \frac{\max\{E_{i,j}, i,j \in \{1,\ldots,k\}\}}{\sum_{i=1}^{k} I_i}$$

The remaining of the procedure described above is not changed. We can show that separation is meaningful, and not a result of over-fitting, by conducting a “permutation test” (see §E.1 in S1 Text).

C.2. **Vectorization.** The following vectorization method is used for both the purpose of unsupervised and supervized classification. The idea is to transform a neuron into a vector that can be easily manipulated for computing distances. Fix a neuron N and let ϕ be a morphological feature. The associated Sholl function ϕ_N has two meaningful values; the area under the curve $s(N) = \int_0^1 |\phi_N(r)| dr$, and either its final value at 1; or its value at the origin $\phi_N(0)$, whichever is more relevant. This number is denoted by $e(N)$ for extreme value. Extreme values are given below for all features that we consider:

- For branching, $e(N)$ is the negative of the number of primary branches.
- For tortuosity, $e(N)$ is the average tortuosity of all branches of N.
- For leaf index, $e(N)$ is the total number of leaves of N.
- For energy, $e(N)$ is representing the total energy at the soma.
- For flux, exceptionally, we pick $e(N)$ to be the flux of the neuron at the first node.
- For taper rate, $e(N)$ is the diameter of the soma.
- For wiring, $e(N)$ is the total dendritic length of the neuron.

In addition to these two values, we also pick two other meaningful values that capture the graph behavior of ϕ_N. Consider all planar points $(r_i, \phi_N(r_i))$, where r_i is the radius of the i-th node. This set of points is approximated by a regression line (least squares) of which equation is $y = a_N x + b_N$. We pick these two values a_N, b_N and add them to our vector. So to each feature ϕ, we obtain a vector

$$(s(N), e(N), a_N, b_N) \tag{29}$$

We concatenate these vectors if we have more than one feature. In our case, we vectorized all descriptors but TMD-Sholl and taper rate. This means that the vector associated to N is in $\mathbb{R}^{24}$.

Remark C.1. We want to highlight that this is just one of possibly many ways to vectorize real-valued functions. This method works well for our purpose here. When our Sholl function happens to have values in more general metric space (as in case of Sholl-TMD that has values in a space of persistence diagrams),

we propose to select a grid of values of the radi and, for each of them, vectorize the given persistence diagram.

C.3. **Dendritic Polarity.** Fix a neuron N. For each node P_i of N, consider the Energy vector $E(P_i) = \dfrac{OP_i}{|OP_i|}$, where O is the Soma, see Eq.(23) in S1 Text. This is a unit vector. Two such vectors $E(P_i)$ and $E(P_j)$, associated to two different nodes, make up an angle whose cosine is the dot product $E(P_i) \cdot E(P_j)$. This angle is small if the vectors point to the same direction, and the angle is large if they do not. We can then consider the "Energy angle matrix"

$$\left[\cos^{-1}(E(P_i) \cdot E(P_j))\right]_{1 \le i \le j \le n} \qquad (30)$$

This is a symmetric matrix with positive coefficients, since all angles are in $[0, \pi]$. A hierarchical clustering is then applied to this matrix and the dendrograms are obtained. Existence of two significantly distinct clusters implies that the neuron is bipolar.

We illustrate this idea on a cortical bipolar cell and the associated hierarchical clustering dendrogram reveals the two clusters corresponding to the two narrow dendritic trees radiating from opposite ends of the soma, see Fig B in S1 Text.

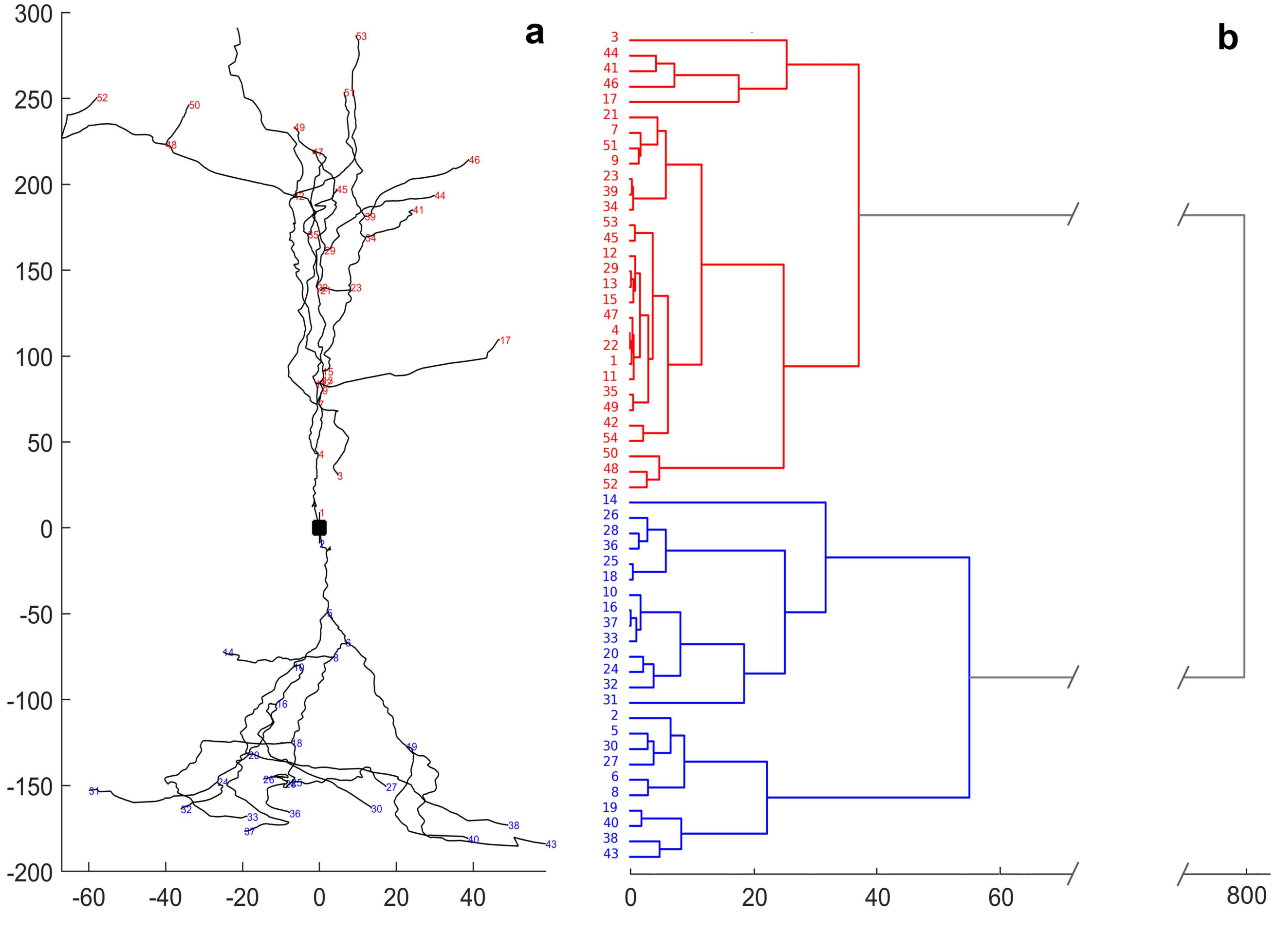

Fig B –Dendrogram of a bipolar interneuron. A representative bipolar cell is shown in **(a)**. The "energy angle matrix" is used to separate the nodes into two clusters as shown in **(b)**. The dendrogram can be read as follows: the angles between pairwise energy vectors associated to red nodes are small as is for the blue nodes. However, the angles between energy vectors of blue and red nodes are much larger.

D. Detection and Feature Selection

Let $C_1, \ldots, C_k$ be k distinct classes of neurons. We say that a descriptor ϕ has at least an $n\%$ level of detection of a class C_i if there is a ball B^ϕ in the d_ϕ metric so that more than $n\%$ of all elements of C_i are within B^ϕ, and of all elements in B^ϕ, more than $n\%$ are from C_i.

Example D.1. Suppose we have three classes of neurons C_1, C_2, C_3, each consisting of 5, 4 and 3 neurons respectively. Let ϕ be a given Sholl descriptor, and suppose there is an ϵ ball in the d_ϕ-metric, that contains 4 elements of C_1 and 2 element from $C_2 \cup C_3$. This ball contains $\frac{4}{5} = 0.8$ of the total of all C_1-neurons (i.e. 80%), while $\frac{4}{6} = 0.66$ or 66% of all neurons in this ball are C_1-neurons. We say that the descriptor ϕ has detected C_1 to a 66% level at least, which is the lower percentage from among 80% and 66%. Fig C in S1 Text illustrates this construction on these three classes.

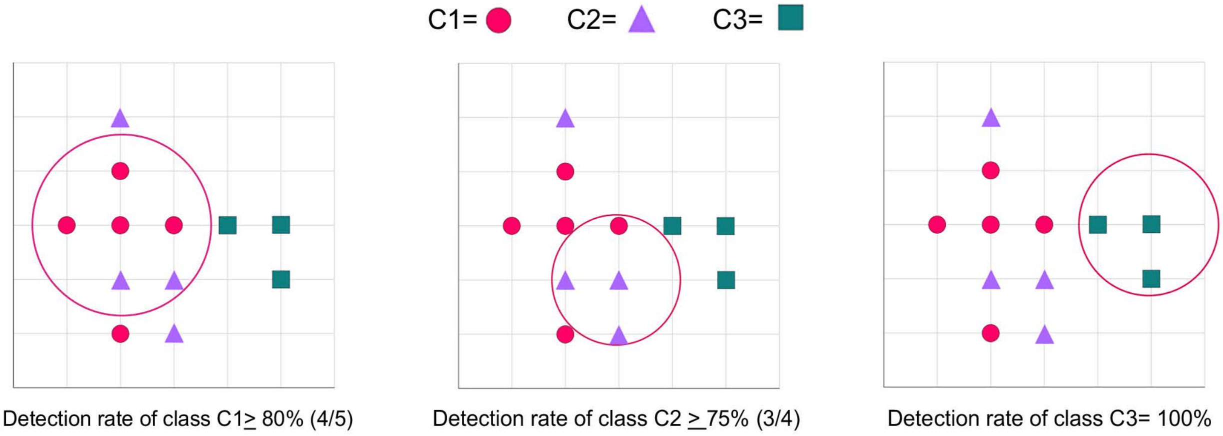

Fig C –Detection rate method. Method used to determine detection rate. Each circle is the boundary of a disk in the Euclidean metric.

We can use detection as a method for feature selection when we run several descriptors on a given set of classes. The set of descriptors with detection rates that are less than a certain percentage are deemed ineffective in differentiating among these classes and can thus be excluded from further analysis (see §E in S1 Text).

We next give details on how to compute the level of detection of a class of neurons, within a collection, by a Sholl descriptor ϕ. Let $\mathcal{N} = \{N_1, \ldots, N_k\}$ be a set of neurons divided up into classes. A neuron N_i will belong to a class $C(N_i)$. Recall that associated to ϕ we have a pseudo-metric d_ϕ on the set of neurons Eq.(2) in S1 Text.

For every neuron $N \in \{N_1, \ldots, N_k\}$, let σ be the permutation of $\{1, \ldots, k\}$ that orders the neurons according to their distance from N, so that

$$0 = d_\phi(N, N_{\sigma(1)}) < d_\phi(N, N_{\sigma(2)}) < \cdots < d_\phi(N, N_{\sigma(k)})$$

- For every $1 \leq i \leq k$, let $det_\phi(N, i)$ denote the smaller of the two numbers:

$$\frac{\#\{N' \in \{N_{\sigma(1)}, N_{\sigma(2)}, \ldots, N_{\sigma(i)}\} \mid C(N) = C(N')\}}{\#C(N)}$$

and

$$\frac{\#\{N' \in \{N_{\sigma(1)}, N_{\sigma(2)}, \ldots, N_{\sigma(i)}\} \mid C(N) \neq C(N')\}}{i}$$

where # means cardinality of set.

- The local detection rate at N, $det_\phi(N)$, is defined as the maximum value of $det_\phi(N, i)$, for $1 \leq i \leq k$.
- The detection rate of a class C is now equal to the maximum of all local detection rates of neurons that belong to that class. Formally

$$det_\phi(C) = \max_{\substack{N \in \mathcal{N} \\ C(N)=C}} det_\phi(N)$$

We suspect the existence of other machine learning procedures that capture the same percentiles. We are not aware of any reference however.

Example D.2. Suppose $\det_\phi(C) = 75\%$. This implies there is a ball in the d_ϕ metric that contains (at least) 75% of all the internal neurons in C, and within that ball, (at least) 75% of all neurons are from C. A detection rate of 100% means perfect detection whereby there is a ball containing all of C and no other neurons from any of the other classes.

E. Metric Learning and Supervized Classification

We use Metric Learning to classify neurons as explained in §4.3 . Given a labeled collection of neurons $\mathcal{N}$ partitioned into k-classes labeled by $\ell_1, \ldots, \ell_k$, we propose to associate to an arbitrary neuron the appropriate class with which it best shares a given number of features.

Firstly, the input collection of neurons is vectorized according to §C.2 in S1 Text. Since each feature whose Sholl function is real-valued $\phi_N : [0,1] \to \mathbb{R}$ contributes 4 entries to this vector, and since we are using 6 features to vectorize, we obtain a vector in $\mathbb{R}^{24}$ for each neuron N. Each vector is therefore given a label since every neuron $N \in \mathcal{N}$ has some label $\ell(N) = \ell_i$ for some i.

Ideally one would hope that the Euclidean metric $d(v_i, v_j) = \sqrt{v_i^T v_j}$ does "separate" the classes, meaning that vectors in the same class are close and those in different classes remain relatively distant. This is hardly the case in practice, so one seeks a modification of this Euclidean metric which has this separation property. A standard approach is to introduce a "matrix of weights" M, which is $k \times k$, positive, so that $D_{ML}(v_i, v_j) = \sqrt{v_i^T M v_j}$ defines a new metric on $\mathbb{R}^n$ (so called Mahalanobis metric, see [1]) with better separating properties with respect to the chosen classes. More precisely, D_{ML} maximizes the sum of distances between points with different labels while keeping the sum of distances between those with similar labels small. Note that since M can be written as LL^T, the associated D_{ML} metric has the following interpretation: it is the distance obtained by first moving vectors via L in $\mathbb{R}^{24}$, then taking their Euclidean distance. This approach is entirely supervised since we need the classes to train the matrix entries and thus the metric.

Our vectors in $\mathbb{R}^{28}$ are the desired input for the Metric Learning procedures implemented for instance in [2]. This uses the Large Margin Nearest Neighbor (LMNN) metric learning algorithm to learn a Mahalanobis distance metric in the K-Nearest Neighbor (KNN) classification setting. this procedure results in a metric D_{ML}, depending on $d_{\phi_1}, \ldots, d_{\phi_k}$, which is our good measure to differentiate between the classes of neurons. There are many ways in which the obtained metric can be used. For instance, given a new neuron N, we may wish to know how close in feature is N to the classes of neurons considered in $\mathcal{N}$. For that purpose, we may apply the following procedure;

- Run metric learning on the vectorized classes of $\mathcal{N}$ to obtain a new metric D_{ML}. The new metric is validated after being tested for "overfitting" (see §E.1 in S1 Text). A good metric gives good separation of the vectorized classes.
- Given a neuron N, vectorize it as in §C.2 in S1 Text and then take its average distances under D_{ML} to the classes in $\mathcal{N}$.
- Pick the class that is, on average, closest to the new neuron N.
- (feature selection) Run each descriptor on the classes. If the detection rates are lower than 80% on all classes, the descriptor can be considered "noisy" and is subsequently excluded. Repeat the process above with non noisy descriptors.

E.1. **Overfitting.** Both grid search §C.1 in S1 Text and metric learning §E in S1 Text provide efficient tools to differentiate classes of neurons. Yet, the fact that these methods return clear separation between two or more classes of trees is not sufficient to conclude that the separation is geometrically meaningful. By itself, those methods may fit exactly against the data.

Example E.1. Let us consider four vertices of a square: $A = (1,1)$, $B = (-1,1)$, $C = (-1,-1)$ and $D = (1,-1)$. Suppose that the first class consists of points A and D, while the second class is composed of points B and C. Metric learning, for instance large margin nearest neighbor algorithm we use, will seek to place elements of different classes far away and those of the same class close together. This can be achieved

by a metric function $d((x_1, y_1), (x_2, y_2)) = \sqrt{A(x_1 - x_2)^2 + a(y_1 - y_2)^2}$ by making A large and a small. Such a perturbed Euclidean metric can be obtained both by the grid search and metric learning. Yet, it is clear that the division to the first and second class is somewhat arbitrary; In fact, putting points A and B to the first class and C and D to the second one is geometrically similar. Alternatively, separation of these classes can be achieved by the same distance function with A small and a large, and therefore can also be found by the methods we present here.

There are two ways an overfitting, as the one described in example E.1 in S1 Text may be detected;

First, when the dataset is large enough, a standard k-fold cross-validation is applied. In this case, a method will be repetitively trained on a subset of the considered dataset, and tested on the remaining test set. Once the results obtained on the test sets are good, we can assume that the methods do not overfit.

In the second case, which is used for small datasets, a procedure similar to a *permutation test* is used. Namely, after obtaining separation of the given classes, we will repetitively permute all the labels of data points and run the grid search / metric learning for the data with the permuted labels. We will check how frequently a good separation between the permuted labels is obtained. If that happens often, then the separation between the initial classes is not valid, as it is rather a result of overfitting. However, of it is not the case, then we have additional evidence that the separation of the original classes is meaningful.

F. Stability

In this final technical section we define stability and then verify that all Sholl descriptors are stable. We address this issue by verifying that our descriptors are reasonably sensitive to small perturbations of input neurons. More precisely, if two reconstructions of the same neuron vary slightly, they will result in different tree representations. A descriptor is "stable" if, when applied to either tree, it gives results that also vary slightly (i.e. the variation is controlled).

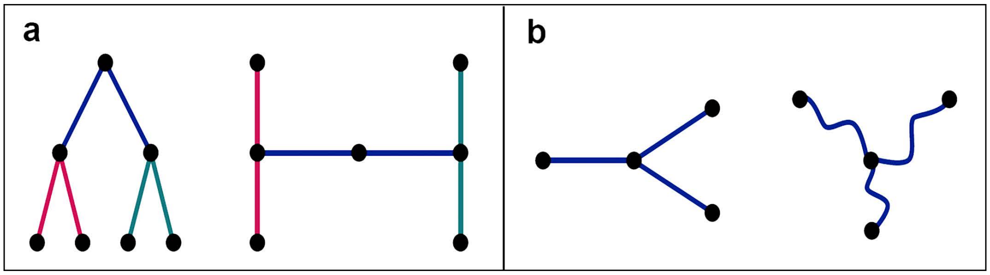

Fig D –Representation of isomorphic trees. Representative isomorphic trees with entirely different (**a**) branching pattern and (**b**) tortuosity descriptors

.

Two different reconstructions of the same neuron produce two different trees embedded in $\mathbb{R}^3$. All reasonable reconstruction schemes should produce isomorphic trees, resulting in the same number of primary dendrites and the same number of bifurcations (Fig D in S1 Text). We can measure the distance between two reconstructed trees under the Hausdorff metric and use it as a measure of closeness. Two such reconstructions are expected to be close in the Hausdorff metric, requiring that our descriptor depends "continuously" on this metric. However, this is not a good notion, as trees that are very close in the Hausdorff metric may still have very different morphological properties (like lengths of branches, number of nodes, etc). Fig E in S1 Text gives an illustration of two trees (**b**) and (**c**) close, in a Hausdorff sense, to the initial tree (**a**) note the tree in (**a**) is represented in gray and overlapping with the tree in (**b**), and is depicted below the main branches in (**c**). Clearly the trees (**b**) and (**c**) have distinct morphological features compared to the tree (**a**) and they should not be considered similar.

Our next definition is adapted from ([3], §2) who utilizes it for rectifiable curves and in the context of knot theory. We will assume that the dendrites are piecewise smooth paths in $\mathbb{R}^3$; meaning that the branches between nodes can be parameterized as C^1-differentiable paths in space.

Let's think of N and N' as two embedded trees in $\mathbb{R}^3$. We say that N and N' are (δ, θ)–close if N' can be obtained from N by a smooth 1-1 map Ψ supported on an open neighborhood of N so that corresponding

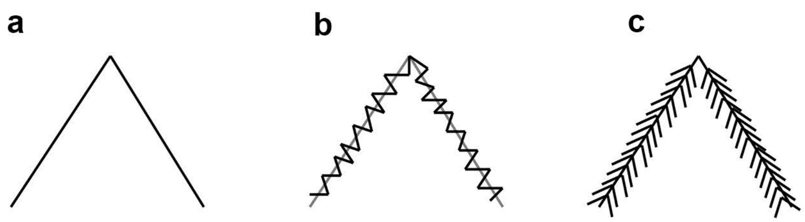

Fig E –Representative trees in the Hausdorff metric. Representative tree (**a**) and similar trees (**b**) and (**c**) that are close to tree (**a**) in the Hausdorff metric.

points x and $\Psi(x)$ are within δ and the norm differences $||\Psi(x) + v - d\Psi_x(v)|| < \theta$ for all $x \in U$ and $v \in T_x\mathbb{R}^3$, where $d\Psi_x$ is the differential of Ψ at x. We recall this is a linear map between tangent spaces $d\Psi_x : T_x\mathbb{R}^3 \to T_{\Psi(x)}\mathbb{R}^3$ mapping a vector v to $\Psi(x) + Jac_x(\Psi)(v)$, where $Jac_x(\Psi)$ is the 3×3 Jacobian matrix of partial derivatives evaluated at x. Let's now make this construction a bit more precise. For the sake of simplicity we will assume Ψ is defined on all of $\mathbb{R}^3$.

Definition F.1. We say that N and N' are (δ, θ)-close if there exists an ambiant diffeomorphism $\Psi : \mathbb{R}^3 \to \mathbb{R}^3$ such that $|\Psi(x) - x| < \delta$ for all x, and the Frobenius norm $||I - Jac_x\Psi|| < \theta$ for every $x \in \mathbb{R}^3$.

Remark F.2. In contrast with the definition in [3], we not only require the angles between corresponding vectors to be close, but also their norms. This is precisely the essence of the inequality $||I - Jac_x\Psi|| < \theta$. Our definition is related to the C^1-topology of functions in the following way. If we view a branch γ of N as a smooth path $[0, 1] \to \mathbb{R}^3$, then it is (δ, θ)-close to $\Psi \circ \gamma$ if both paths are C^1-close.

We now define "stability". Recall that in defining our descriptors, we viewed a neuron as an embedded tree in $\mathbb{R}^3$, up to $O(3)$-isometry. Our definition of stability should apply to isometry classes of neurons. Let $[N]$ be the isometry class of N, which means that $[N]$ is the orbit of N under the action of $O(3)$, i.e. $[N] = \{AN, A \in O(3)\}$. We say that $[N]$ and $[N']$ are (δ, θ)-close if there are representatives $N_1 \in [N]$ and $N_1' \in [N']$ that are (δ, θ) close as in Definition §F.3 in S1 Text. To ease notation, we remove the bracket notation and say that N and N' are (ϵ, θ)-close if they have chosen representatives, also denoted by N and N', which are (ϵ, θ) close in $\mathbb{R}^3$.

Definition F.3. A Sholl descriptor ϕ is *stable* if for any $\epsilon > 0$, there exists $\eta > 0$ so that for $\delta < \eta, \theta < \eta$,

$$N \text{ and } N' \text{ are } (\delta, \theta)\text{-close} \implies d_\phi(N, N') < \epsilon$$

According to this definition, a small perturbation or deformation of the neuron which "moves the points by as little as δ" and "distorts the angles by as little as θ", yields a small change in the descriptor ϕ.

Let N be a neuron represented as a spatial tree, and let ϕ be a Sholl descriptor. The nodes for N are sorted according to increasing distances from the soma $0 < r_1 < \cdots < r_k$, with k being the number of nodes. These distances are radial or dendritic depending on the descriptor. We make the assumption that a deformation of a neuron does not introduce new bifurcations, and so the leaf index is completely unchanged by deformation. It is evidently stable.

We start by verifying the stability of the branching pattern descriptor. This descriptor is only based on the distribution of nodes, and so only the δ constant matters. The nodes have (normalized) distances from the soma $r_i \leq 1$. Let's move the nodes of N by a distance δ, and by that we mean there is a homeomorphism $\Psi : N \to N'$, taking node P_i to P_i' so that for all i, $|r_i - r_i'| \leq \delta$. Note that we start with N that is normalized (i.e. it is within a ball of radius 1) but then N' might not be. By normalizing N' to N'', and taking the new node radii $r_i'' \leq 1$, we see that

$$|r_i - r_i''| \leq |r_i - r_i'| + |r_i' - r_i''| \leq |r_i - r_i'| + \left|\left(1 - \frac{1}{1+\delta}\right) r_i'\right| \leq |r_i - r_i'| + \delta$$

So we can assume from the start that both nodes of N and N' are such that $r_i, r_i' \leq 1$, and that for all i, $|r_i - r_i'| \leq 2\delta$. Recall next that the t_i's are a renaming of the r_i and r_j', $1 \leq i, j \leq k$, once they are

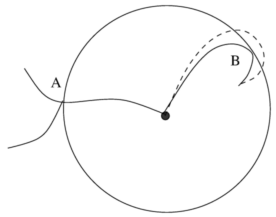

Fig F –Instability. Instability behavior for tortuosity descriptor.

ordered by increasing order, so that $t_1 < t_2 < \ldots < t_{2k}$ (see Eq.(6) in S1 Text). Given this, the formula for functional distance takes the form

$$d_\phi(N, N') = d^{L^1}(\phi_N, \phi_{N'}) = \sum_{i=0}^{2k-1} (t_{i+1} - t_i)|\phi_N(t_i) - \phi_{N'}(t_i)|$$

Choose $\delta < \min_i\{|r_i - r_{i+1}|\}$. By construction of the branching pattern, the difference $\phi_N(t_i) - \phi_{N'}(t_i)$ can be at most ± 1 on intervals $[t_i, t_{i+1}[$ of the form $[r_j, r'_{j+1}[$ or $[r'_j, r_{j+1}[$ for some j (j need not be same as i since this is obtained after renumbering), and is zero otherwise. These intervals are of length 2δ or less. This gives that $d_\phi(N, N') \leq 2k \cdot 2\delta = 4k\delta$, where k is the number of nodes. By choosing δ small enough, this is less than any desired ϵ.

The taper and energy descriptors depend only on the node distribution, and are thus stable. Note that for the taper rate, we assume that the width of a dendrite at a given node is the same in any reconstruction (this is not a varying feature in our definition of stability), so this descriptor only depends on nodes, and it is stable.

To see stability of the TMD with the radial distance to the soma let us observe that the TMD-path decomposition may change when the position of nodes is perturbed. This will happen when the endpoints of two paths that merge at a bifurcation point b are at almost the same distance from the soma. In this case a perturbation of the endpoints of those paths may result in swapping the branch that continues up from b with the one that terminates there. However, since the TMD only gathers the values of distances from the soma, the endpoints of persistence intervals will move by at most δ which directly translate in stability of the descriptor.

As for the Tortuosity descriptor, we recall that T associates to every N and $r \in [0, 1]$ the average tortuosity of N_r which is the connected component of N containing the soma inside the ball of radius r. The stability for T holds under one condition, and so we refer to it as "conditional stability". We discuss this condition next and remark that it is almost always realized so that in practice and generically T behaves stably. To understand this condition, we must observe that instability can occur in the following situation illustrated in Fig F in S1 Text.

Here r is chosen to be the radial distance of the node A. The twisted dendrite touches the sphere tangentially at B. When measuring tortuosity of N_r, we only consider the term δ_{SB} Eq.(12) in S1 Text which is the tortuosity from the soma S to B considered as a leaf. If a small perturbation causes the branch to be inside the sphere, this term is replaced by the term δ_{SC} which is the tortuosity of the entire branch inside $B(r)$ from S to C. This leads to a sudden increase in tortuosity which can potentially lead to instability. However this only happens if indeed a sphere through a node is tangent to a branch, which is a rare instance.

Let $\gamma : [0, 1] \longrightarrow \mathbb{R}^3$ be a smooth space curve. Then its length is given by $\int_0^1 |\gamma'(t)|dt$. We say that the two paths γ_1 and γ_2 are (δ, θ) close if there is a (δ, θ)-diffeomorphism taking γ_1 to γ_2; that is $\gamma_2 := \Psi \circ \gamma_1$.

Lemma F.4. *Let γ_1 be a given smooth curve in $\mathbb{R}^3$. For every $\epsilon > 0$, $\exists \eta$ with $\delta < \eta, \theta < \eta$, such that for every curve γ_2 that is (δ, θ)-close to γ_1,*

$$\left| \int_0^1 |\gamma_2'(t)| - |\gamma_1'(t)| dt \right| < \epsilon$$

Proof. Let γ_2 be (δ,θ)-close to γ_1, meaning there is a (δ,θ)-diffeomorphism Ψ taking γ_1 to γ_2. Since γ_1 is differentiable, $|\gamma_1'(t)|$ is bounded uniformly on $[0,1]$, say by $M>0$. We can write

$$\begin{aligned}
\left|\int_0^1 |\gamma_1'(t)| - |\gamma_2'(t)| dt\right| &\leq \int_0^1 ||\gamma_1'(t)| - |\gamma_2'(t)||dt \\
&\leq \int_0^1 |\gamma_1'(t) - \gamma_2'(t)|dt \\
&\leq \int_0^1 |\gamma_1'(t) - Jac_t\Psi(\gamma_1'(t)|dt \quad \text{here } Jac_t\Psi := Jac_{\gamma_1(t)}\Psi \\
&= \int_0^1 \left|(I - Jac_t\Psi)(\gamma_1'(t)\right| dt \\
&\leq M\int_0^1 ||I - Jac_t\Psi||dt \leq M\vartheta
\end{aligned}$$

where we know by definition that $||I - Jac_t\Psi|| \leq \theta$ on the domain of Ψ. By choosing $\eta = \frac{\epsilon}{M}$, we obtain our claim. □

Let Ψ be (δ,θ)-diffeomorphism taking N to N'. The map Ψ maps nodes to nodes, branches to branches necessarily. Lemma F.4 in S1 Text shows that by controlling (δ,θ) we can control the lengths of branches. It is clear that T is stable away from the stated condition, meaning that if spheres through the nodes of N and N' are not tangent to dendrites, then $d_T(N,N') = d_T(N,\Psi(N)) < \epsilon$ for any chosen $\epsilon > 0$, once δ,θ are chosen sufficiently small. We skip the details.

Finally we discuss the stability of the flux descriptor. The stability in these cases hinges on controlling the variation of angles in any neuron deformation. This is a direct consequence of the following lemma.

Lemma F.5. *Let $v_1, v_2 \in T_x\mathbb{R}^3$ be unit vectors, $x \in N$ (x will be typically a node in our case). Then for any $\epsilon > 0$, $\exists \eta > 0$ such that for any (δ,θ)-diffeomorphism Ψ with $\theta < \eta$,*

$$|\angle(v_1,v_2) - \angle(d\Psi_x(v_1), d\Psi_x(v_2))| < \epsilon$$

Proof. We fix x and drop it from the notation. We write $d_u\Psi(v_i) = \dfrac{d\Psi(v_i)}{|d\Psi(v_i)|}$ the normalized vector. Using the cosine-angle formula $|v_1 - v_2|^2 = |v_1|^2 + |v_2|^2 - 2|v_1||v_2|\cos\angle(v_1,v_2)$, we can write

$$|d_u\Psi(v_1) - d_u\Psi(v_2)|^2 = 2 - 2\cos\angle(d_u\Psi(v_1), d_u\Psi(v_2))$$
$$|v_1 - v_2|^2 = 2 - 2\cos\angle(v_1,v_2)$$

By taking the difference, we see immediately that we can make the angles $\angle(v_1,v_2)$ and $\angle(d\Psi_x(v_1), d\Psi_x(v_2))$ arbitrarily close by making their cosines arbitrarily close or equivalently by making $|d_u\Psi(v_1) - d_u\Psi(v_2)|$ and $|v_1 - v_2|$ arbitrarily close. We check this last part.

$$\begin{aligned}
&||d_u\Psi(v_1) - d_u\Psi(v_2)| - |v_1 - v_2|| \\
\leq\ & |d_u\Psi(v_1) - v_1| + |d_u\Psi(v_2) - v_2| \\
=\ & \left|d\Psi\left(\frac{v_1}{|d\Psi(v_1)|}\right) - v_1\right| + \left|d\Psi\left(\frac{v_2}{|d\Psi(v_2)|}\right) - v_2\right| \quad \text{by linearity of } d\Psi \\
\leq\ & \left|d\Psi\left(\frac{v_1}{|d\Psi(v_1)|}\right) - \frac{v_1}{|d\Psi(v_1)|}\right| + \left|d\Psi\left(\frac{v_2}{|d\Psi(v_2)|}\right) - \frac{v_2}{|d\Psi(v_2)|}\right| + \left|\frac{v_1}{|d\Psi(v_1)|} - v_1\right| + \left|\frac{v_2}{|d\Psi(v_2)|} - v_2\right|
\end{aligned}$$

Since for any vector $v \in T_x\mathbb{R}^3$, $|d\Psi(v) - v| < \theta$, we can extend the string of inequalities above to

$$\begin{aligned}
||d_u\Psi(v_1) - d_u\Psi(v_2)| - |v_1 - v_2|| &\leq 2\theta + \frac{|1 - |d\Psi(v_1)||}{|d\Psi(v_1)|} + \frac{|1 - |d\Psi(v_2)||}{|d\Psi(v_2)|} \\
&\leq 2\theta + 2\frac{\theta}{1-\theta}
\end{aligned}$$

By making θ small, the left term can be made arbitrarily small and this is enough to yield our claim. □

References

1. Bellet A, Habrard A, Sebban M. Metric Learning. Morgan and Claypool Publishers; 2015.
2. de Vazelhes W, Carey CJ, Tang Y, Vauquier N, Bellet A. metric-learn: Metric Learning Algorithms in Python. Journal of Machine Learning Research. 2020;21:1–6. doi:10.48550/arXiv.1908.04710.
3. Denne E, Sullivan JM. Convergence and Isotopy Type for Graphs of Finite Total Curvature. Geometric Topology. 2008; p. 163–174. doi:10.1007/978-3-7643-8621-4_8.
